# Asthma Surveillance — United States, 2006–2018

**DOI:** 10.15585/mmwr.ss7005a1

**Published:** 2021-09-17

**Authors:** Cynthia A. Pate, Hatice S. Zahran, Xiaoting Qin, Carol Johnson, Erik Hummelman, Josephine Malilay

**Affiliations:** 1 Division of Environmental Health Science and Practice, National Center for Environmental Health, CDC

## Abstract

**Problem:**

Asthma is a chronic disease of the airways that requires ongoing medical management. Socioeconomic and demographic factors as well as health care use might influence health patterns in urban and rural areas. Persons living in rural areas tend to have less access to health care and health resources and worse health outcomes. Characterizing asthma indicators (i.e., prevalence of current asthma, asthma attacks, emergency department and urgent care center [ED/UCC] visits, and asthma-associated deaths) and determining how asthma exacerbations and health care use vary across the United States by geographic area, including differences between urban and rural areas, and by sociodemographic factors can help identify subpopulations at risk for asthma-related complications.

**Reporting Period:**

2006–2018.

**Description of System:**

The National Health Interview Survey (NHIS) is an annual cross-sectional household health survey among the civilian noninstitutionalized population in the United States. NHIS data were used to produce estimates for current asthma and among them, asthma attacks and ED/UCC visits. National Vital Statistics System (NVSS) data were used to estimate asthma deaths. Estimates of current asthma, asthma attacks, ED/UCC visits, and asthma mortality rates are described by demographic characteristics, poverty level (except for deaths), and geographic area for 2016–2018. Trends in asthma indicators by metropolitan statistical area (MSA) category for 2006–2018 were determined. Current asthma and asthma attack prevalence are provided by MSA category and state for 2016–2018. Detailed urban-rural classifications (six levels) were determined by merging 2013 National Center for Health Statistics (NCHS) urban-rural classification data with 2016–2018 NHIS data by county and state variables. All subregional estimates were accessed through the NCHS Research Data Center.

**Results:**

Current asthma was higher among boys aged <18 years, women aged ≥18 years, non-Hispanic Black (Black) persons, non-Hispanic multiple-race (multiple-race) persons, and Puerto Rican persons. Asthma attacks were more prevalent among children, females, and multiple-race persons. ED/UCC visits were more prevalent among children, women aged ≥18 years, and all racial and ethnic groups (i.e., Black, non-Hispanic Asian, multiple race, and Hispanic, including Puerto Rican, Mexican, and other Hispanic) except American Indian and Alaska Native persons compared with non-Hispanic White (White) persons. Asthma deaths were higher among adults, females, and Black persons. All pertinent asthma outcomes were also more prevalent among persons with low family incomes. Current asthma prevalence was higher in the Northeast than in the South and the West, particularly in small MSA areas. The prevalence was also higher in small and medium metropolitan areas than in large central metropolitan areas. The prevalence of asthma attacks differed by MSA category in four states. The prevalence of ED/UCC visits was higher in the South than the Northeast and the Midwest and was also higher in large central metropolitan areas than in micropolitan and noncore areas. The asthma mortality rate was highest in non-MSAs, specifically noncore areas. The asthma mortality rate was also higher in the Northeast, Midwest, and West than in the South. Within large MSAs, asthma deaths were higher in the Northeast and Midwest than the South and West.

**Interpretation:**

Despite some improvements in asthma outcomes over time, the findings from this report indicate that disparities in asthma indicators persist by demographic characteristics, poverty level, and geographic location.

**Public Health Action:**

Disparities in asthma outcomes and health care use in rural and urban populations identified from NHIS and NVSS can aid public health programs in directing resources and interventions to improve asthma outcomes. These data also can be used to develop strategic goals and achieve CDC’s Controlling Childhood Asthma and Reducing Emergencies (CCARE) initiative to reduce childhood asthma hospitalizations and ED visits and prevent 500,000 asthma-related hospitalizations and ED visits by 2024.

## Introduction

Asthma is a chronic respiratory disease requiring ongoing medical management. In 2017, asthma resulted in an estimated 1.6 million emergency department (ED) visits and 183,000 hospitalizations in the United States (*1*). Asthma has had a considerable economic impact (*2*) and resulted in a substantial number of missed school days (*3*,*4*). In the United States, nearly 24.8 million persons (7.7% of the population) had current asthma in 2018 (*1*). Among children (persons aged <18 years), asthma was more prevalent among those in families with low incomes and among non-Hispanic Black (Black) children and those of Puerto Rican descent compared with non-Hispanic White (White) children (*5*). Approximately, one half of persons with current asthma reported having had an asthma attack within the past 12 months (*1*). Asthma has been associated with substantial morbidity and remains a focus of the U.S. Department of Health and Human Services (HHS) *Healthy People 2030* initiative (*6*,*7*).

Geographic disparities (e.g., regional, urban, and rural) in health outcomes have been documented (*8*–*10*), with higher numbers of excess deaths from chronic lower respiratory disease in rural areas than in urban areas (*11*). Socioeconomic and demographic factors (e.g., poverty, education status, age, race/ethnicity, sex, and insurance status) and health care use might contribute to health outcome patterns observed in urban and rural areas (*9*,*12*–*14*). Persons living in rural areas typically have worse health outcomes and less access to health care than those in urban areas (*8*,*9*,*12*,*15*). Recent studies have focused attention on rural health and related issues (*16*,*17*). Rural residents are more likely to live in areas with hospital closures (*18*), travel long distances to receive specialty or emergency care, and live in areas with shortages in the health care workforce, subspecialty care, and preventative services; they are also less likely to have health insurance (*8*,*9*). Barriers to health care access can result in unmet health care needs and preventable hospitalizations (*19*). Among inner-city residents, risk factors for developing asthma and experiencing asthma exacerbations include mold or mildew in homes (*20*), air pollution, lack of access to health care, and exposure to secondhand smoke (*21*). In contrast, suburban residents have been shown to have the best health outcomes (*8*).

Social determinants of health play a substantial role in health outcomes (*10*). Reducing racial and ethnic disparities in asthma risks and health care is of national strategic importance (*19*,*22*,*23*). Asthma prevalence disparities among various racial/ethnic groups increased during 1999–2011 (*24*). Children, Black persons, Hispanic persons, persons insured by Medicaid or the Children’s Health Insurance Program, and persons living in the Northeast are more likely to visit an ED for asthma (*25*), which is a key indicator of poorly controlled asthma (*26*). Racial/ethnic health disparities among rural adults aged ≥18 years also have been reported (*12*). Limited health care access and unmet health care needs can result in serious life-threatening respiratory episodes and hinder timely access to ED care and survival (*15*).

CDC analyzed 2006–2018 data from the National Health Interview Survey (NHIS) to determine the prevalence of current asthma, asthma attacks, asthma-related ED and urgent care center (UCC) visits, and deaths for which asthma was the underlying cause by certain demographic characteristics, poverty level, and geographic location in the United States. This report is fifth in a series of asthma surveillance summaries (*27*–*30*) with a focus on geographic areas, including states, metropolitan statistical area (MSA) category, and six-level urban-rural classification.

The findings from this report can be used by National Asthma Control Program (NACP) funding recipients, public health services, asthma programs, and health care providers to direct interventions, strategic activities, and resource allocations toward specific sociodemographic groups and geographic locations to reduce asthma-related adverse health outcomes and premature deaths. These measures can be implemented with the aid of evidence-based strategies in the Exhale Technical Package (*31*) to support the Controlling Childhood Asthma and Reducing Emergencies (CCARE) goal of preventing 500,000 asthma-related hospitalizations and ED visits by 2024 (*32*).

## Methods

### Data Source 

NHIS is a cross-sectional, household (in-person) health survey of the civilian noninstitutionalized population in the United States (*33*). The final response rate in 2018 was 59.2% for children and 53.1% for adults (*33*). NHIS data from 2006–2018 were analyzed to estimate trends in the prevalence of current asthma, asthma attacks, and asthma-related health care visits by MSA category. These asthma indicators were further analyzed by demographics, poverty level (except for deaths), and geographic area using combined years 2016–2018. Analyses for geographic areas smaller than U.S. Census region (e.g., state, MSA category, and urban-rural classification) were accessed through the National Center for Health Statistics (NCHS) Research Data Center. Data for all other variables were analyzed using publicly available data. Poverty level was defined by the ratio of family income to the federal poverty threshold (i.e., ratio of income to poverty). Respondents were considered to have current asthma if they answered “yes” to the questions, “Have you ever been told by a doctor or other health professional that you had asthma?” and “Do you still have asthma?” Respondents were considered to have had an asthma attack in the past year if they answered “yes” to the question, “During the past 12 months, have you had an episode of asthma or an asthma attack?” Respondents were considered to have had an asthma-related ED/UCC visit in the past year if they answered “yes” to the question, “During the past 12 months, have you had to visit an emergency room or urgent care center because of asthma?” (*33*). Asthma mortality rates were obtained from the NCHS National Vital Statistics System (NVSS), accessed through the CDC WONDER online tool (*34*) with asthma as underlying cause of death, using *International Classification of Diseases, Tenth Revision* codes J45 and J46.

### Description of Variables 

Demographic characteristics included sex (male or female), age group (0–4 years, 5–11 years, 12–17 years, 18–24 years, 25–34 years, 35–64 years, and ≥65 years), race and ethnicity (White, Black, non-Hispanic American Indian or Alaska Native [AI/AN], non-Hispanic Asian [Asian], non-Hispanic multiple-race [multiple-race], and Hispanic), ethnicity subgroup (Hispanic, including Puerto Rican, Mexican, and other Hispanic, and non-Hispanic), and poverty level. Using five publicly available imputed income files, the ratio of income to poverty was categorized into four federal poverty levels (FPLs) (<100% FPL, 100% to <250% FPL, 250% to <450% FPL, and ≥450% FPL). The U.S. Census Bureau’s federal poverty threshold is based on family income and family size. Mortality data could not be analyzed by income status because death certificates on which the mortality data are based do not include information on income (*34*). Geographic area variables include U.S. Census region (Northeast, Midwest, South, and West), U.S. Census division (New England, Middle Atlantic, East North Central, West North Central, South Atlantic, East South Central, West South Central, Mountain, and Pacific), state (2010 U.S. Census Federal Information Processing Standard state code), MSA categories (large MSA: population ≥1 million; small MSA: population <1 million; and non-MSA: persons not living in an MSA), and urban-rural classification (large central metropolitan, large fringe metropolitan, medium metropolitan, small metropolitan, micropolitan, and noncore). Results by U.S. Census division, demographics, and poverty level by MSA categories are provided (Supplementary Tables, https://stacks.cdc.gov/view/cdc/109086). State and county variables were merged with the 2013 NCHS Urban–Rural Classification Scheme for Counties (*35*) using restricted-use NHIS data at the NCHS Research Data Center to provide a six-level urban-rural classification. Subcategories from most urban to most rural include 1) large central metropolitan (counties in MSAs of ≥1 million population containing the principal city), 2) large fringe metropolitan (counties in MSAs of ≥1 million population not containing the principal city), 3) medium metropolitan (counties in MSAs of 250,000–999,999 population), 4) small metropolitan (counties in MSAs of <250,000 population), 5) micropolitan (urban cluster population of 10,000–49,999), and 6) noncore (nonmetropolitan counties that did not qualify as micropolitan, including those without an urban cluster population of at least 10,000).

### Statistical Analysis

NHIS data for 2016–2018 were combined to obtain sufficient sample sizes for analysis of subregional geographic area estimates, including U.S. Census division, MSA categories, urban-rural classification, and state. Trends were analyzed for asthma indicators (i.e., prevalence of current asthma, asthma attacks, ED/UCC visits, and asthma-associated deaths) across annual years 2006–2018. NHIS data by MSA category were stratified by state, and samples were weighted to adjust for nonresponse, poststratification, and probability of selection (*33*). The percentages and standard errors were calculated using SAS (version 9.4; SAS Institute) and SAS-callable SUDAAN (version 11; Research Triangle Institute) to account for survey’s complex sample design.

Prevalence of current asthma, asthma attacks, ED/UCC visits, and mortality rates are presented by demographic characteristics, poverty level (except for deaths), U.S. Census region, MSA category, six-level urban-rural classification, and state (for current asthma and asthma attacks only). State-level estimates of current asthma and asthma attacks by MSA category also are provided. Unadjusted estimates are used to present prevalence estimates as observed to be consistent with the previous report (*30*). All other results are included elsewhere (Supplementary Tables, https://stacks.cdc.gov/view/cdc/109086). Associations between asthma indicators (except deaths) by demographic characteristics, poverty level, and geographic variables were assessed using chi-square tests. Nondirectional two-tailed Z-tests were used to determine the statistical significance of differences between two percentages. Joinpoint statistical software (version 4.8.0.1; National Cancer Institute) was used to determine the statistical significance of trends (*36*). Statistical significance was set at p<0.05. 

## Results

### Prevalence of Current Asthma 

**FIGURE 1:**
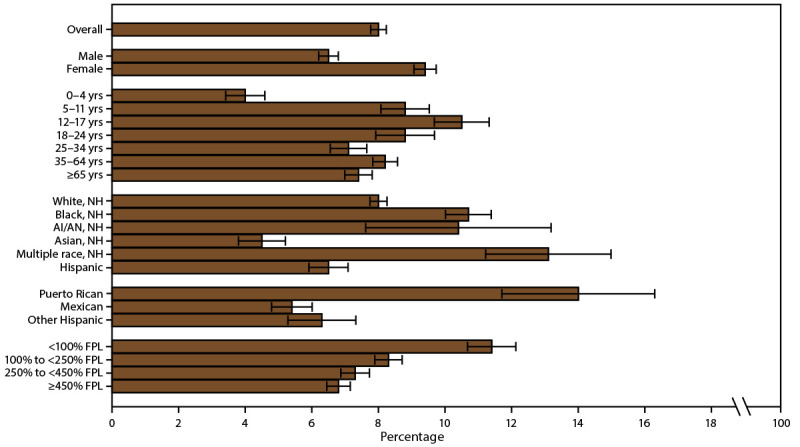
Current asthma* prevalence,^†^ by sex, age group, race/ethnicity,^§^ and federal poverty level^¶^ — United States, 2016–2018 **Source:** CDC, National Center for Health Statistics, National Health Interview Survey. https://www.cdc.gov/nchs/nhis/index.htm **Abbreviations:** AI/AN = American Indian or Alaska Native; FPL = federal poverty level; NH = non-Hispanic. * Includes persons who answered “yes” to the questions, “Have you ever been told by a doctor or other health professional that you had asthma?” and “Do you still have asthma?” ^†^ Prevalence is the proportion of the population who reported having current asthma, with 95% confidence intervals indicated by error bars. ^§^ Puerto Rican, Mexican, and other Hispanic are subsets of Hispanic. ^¶^ Determined by family income and size using U.S. Census Bureau poverty thresholds. Poverty level is defined as the ratio of family income to federal poverty threshold in terms of FPL.

During 2016–2018, approximately 8.0% of the U.S. population reported having current asthma, with 8.1% among children aged 0–17 years and 7.9% among adults aged ≥18 years (Table 1). The percentages of persons with current asthma stratified by age group are provided (Figure 1).

**TABLE 1:** Characteristics of persons of all ages and prevalence of those with current asthma* among all ages, children aged 0–17 years, and adults aged ≥18 years, by geographic area — United States, 2016–2018

Characteristic	All ages			Current asthma								
				All ages			Children			Adults		
	Weighted no.^†^	% (SE)	(95% CI)	Weighted no.^†^	Prevalence % (SE)	(95% CI)	Weighted no.^†^	Prevalence % (SE)	(95% CI)	Weighted no.^†^	Prevalence % (SE)	(95% CI)
**Total**	**320,600,934**	**—**	**—**	**25,486,467**	**8.0 (0.12)**	**7.7–8.2**	**5,947,939**	**8.1 (0.55)**	**7.7–8.6**	**19,538,528**	**7.9 (0.13)**	**7.7–8.2**
**U.S. Census region**	—	—	—	p = 0.003^§^			p = 0.01^§^			p = 0.001^§^		
Northeast	56,983,341	17.8 (0.73)	(16.4–19.3)	5,050,436	8.9 (0.31)	(8.3–9.5)	1,142,095	9.1 (0.76)	(7.8–10.7)	3,908,341	8.8 (0.33)	(8.2–9.5)
Midwest	69,891,347	21.8 (0.63)	(20.6–23.1)	5,669,742	8.1 (0.22)	(7.7–8.6)	1,243,798	8.0 (0.41)	(7.2–8.8)	4,425,944	8.2 (0.23)	(7.7–8.6)
South	117,084,584	36.5 (1.01)	(34.6–38.5)	8,902,468	7.6 (0.19)	(7.2–8.0)	2,324,759	8.5 (0.37)	(7.8–9.2)	6,577,709	7.4 (0.21)	(7.0–7.8)
West	76,641,661	23.9 (0.94)	(22.1–25.8)	5,863,821	7.7 (0.28)	(7.1–8.2)	1,237,288	6.9 (0.38)	(6.2–7.7)	4,626,534	7.9 (0.31)	(7.3–8.5)
**MSA category^¶^**	—	—	—	p = 0.009^§^			p = 0.10			p = 0.04^§^		
Large MSA	182,494,015	56.9 (0.74)	(55.5–58.4)	13,981,756	7.7 (0.15)	(7.4–8.0)	3,242,332	7.7 (0.28)	(7.2–8.3)	10,739,424	7.7 (0.16)	(7.4–8.0)
Small MSA	96,095,562	30.0 (1.25)	(27.6–32.5)	8,082,693	8.4 (0.20)	(8.0–8.8)	1,948,888	8.7 (0.39)	(8.0–9.5)	6,133,805	8.3 (0.22)	(7.9–8.8)
Non-MSA	42,011,357	13.1 (1.10)	(11.1–15.4)	3,422,018	8.2 (0.31)	(7.6–8.8)	756,719	8.3 (0.59)	(7.2–9.5)	2,665,299	8.1 (0.32)	(7.5–8.8)
Urban-rural classification**	—	—	—	p = 0.002^§^			p = 0.25			p = 0.005^§^		
Large central metropolitan	100,582,672	31.4 (0.49)	(30.4–32.3)	7,327,448	7.3 (0.19)	(6.9–7.7)	1,700,141	7.6 (0.38)	(6.9–8.3)	5,627,307	7.2 (0.21)	(6.8–7.6)
Large fringe metropolitan	82,685,805	25.8 (0.60)	(24.6–27.0)	6,705,839	8.1 (0.23)	(7.7–8.6)	1,546,829	7.9 (0.42)	(7.1–8.7)	5,159,010	8.2 (0.26)	(7.7–8.7)
Medium metropolitan	65,861,315	20.5 (1.17)	(18.3–22.9)	5,558,467	8.5 (0.25)	(8.0–9.0)	1,381,782	9.0 (0.46)	(8.2–10.0)	4,176,685	8.3 (0.28)	(7.7–8.8)
Small metropolitan	29,459,786	9.2 (1.10)	(7.2–11.6)	2,472,695	8.4 (0.33)	(7.8–9.1)	562,468	8.3 (0.75)	(6.9–9.9)	1,910,227	8.4 (0.35)	(7.8–9.1)
Micropolitan	26,461,517	8.3 (0.97)	(6.5–10.4)	2,128,466	8.1 (0.39)	(7.3–8.9)	470,805	8.2 (0.77)	(6.8–9.8)	1,657,661	8.0 (0.41)	(7.3–8.9)
Noncore	15,549,840	4.9 (0.67)	(3.7–6.3)	1,293,551	8.3 (0.47)	(7.4–9.3)	285,914	8.4 (1.00)	(6.6–10.6)	1,007,638	8.3 (0.50)	(7.4–9.3)

**Source:** CDC, National Center for Health Statistics, National Health Interview Survey. https://www.cdc.gov/nchs/nhis/index.htm

**Abbreviations:** CI = confidence interval; MSA = metropolitan statistical area; SE = standard error.

* Includes persons who answered “yes” to the questions, “Have you ever been told by a doctor or other health professional that you had asthma?” and “Do you still have asthma?”

^†^ National Health Interview Survey sample weights were used to adjust for nonresponse, poststratification, and probability of selection to provide estimates for the intended U.S. populations.

^§^ Statistically significant by chi-square test, p<0.05.

^¶^ Large MSAs have a population of ≥1 million; small MSAs have a population of <1 million. Non-MSAs consist of persons not living in an MSA.

** Large central metropolitan areas are counties in MSAs of ≥1 million population containing the principal city; large fringe metropolitan areas are counties in MSAs of ≥1 million population not containing the principal city; medium metropolitan areas are counties in MSAs of 250,000–999,999 population; small metropolitan areas are counties in MSAs of <250,000 population; micropolitan areas have an urban cluster population of 10,000–49,999; and noncore areas include nonmetropolitan areas that did not qualify as micropolitan, including those without an urban cluster population of at least 10,000.

#### Trends in Prevalence of Current Asthma 

**FIGURE 2:**
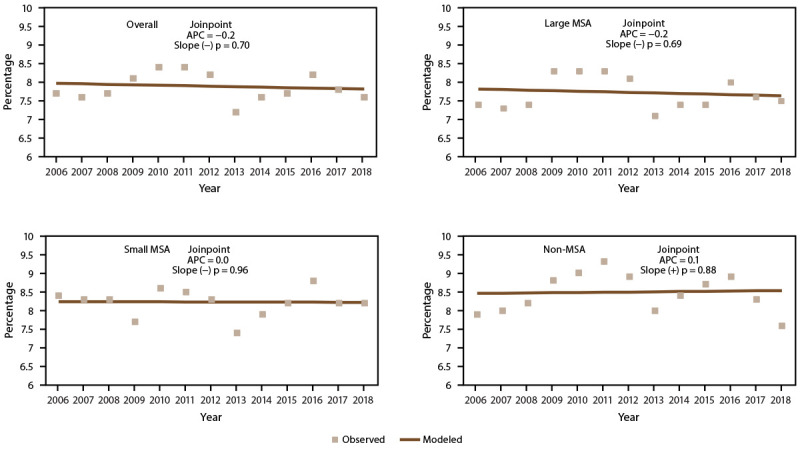
Current asthma* prevalence,^†^ by metropolitan statistical area category^§^ — United States, 2006–2018 **Source:** CDC, National Center for Health Statistics, National Health Interview Survey. https://www.cdc.gov/nchs/nhis/index.htm **Abbreviations:** APC = annual percent change; MSA = metropolitan statistical area. * Includes persons who answered “yes” to the questions, “Have you ever been told by a doctor or other health professional that you had asthma?” and “Do you still have asthma?” ^†^ Prevalence is the proportion of the population who reported having current asthma. Significance set at p<0.05. ^§^ Large MSAs have a population of ≥1 million; small MSAs have a population of <1 million. Non-MSAs consist of persons not living in an MSA.

During 2006–2018, overall and by MSA category, current asthma prevalence among all ages (Figure 2) and adults did not change significantly (Supplementary Tables 3 and 23, https://stacks.cdc.gov/view/cdc/109086). However, among children, a decrease occurred in the overall trend, not considering MSA categories, in asthma prevalence (annual percent change [APC] = −1.6) and the trend in small MSAs (APC = −1.5). In addition, during 2011–2018, asthma prevalence decreased among children in large MSAs (APC = −4.0).

#### Prevalence of Current Asthma by Demographic Characteristics and Poverty Level

Current asthma prevalence differed by demographic characteristics, regardless of MSA category (Supplementary Table 2, https://stacks.cdc.gov/view/cdc/109086). Current asthma prevalence was 8.1% among children, 7.9% among adults (Supplementary Table 1, https://stacks.cdc.gov/view/cdc/109086), 6.5% among males, and 9.4% among females (Figure 1) (Supplementary Table 1, https://stacks.cdc.gov/view/cdc/109086). The difference in asthma prevalence between males and females varied by age group. Among children, boys had a higher prevalence (9.0%) than girls (7.1%), whereas among adults, women had a higher prevalence (10.0%) than men (5.7%) (Supplementary Table 1, https://stacks.cdc.gov/view/cdc/109086). Current asthma prevalence was lowest among children aged 0–4 years (4.0%) and highest among those aged 12–17 years (10.5%). Among all ages, prevalence was higher among Black persons (10.7%) and persons of multiple races (13.1%) and lower among Asian (4.5%) and Hispanic persons (6.5%) than among White persons (8.0%) (Figure 1) (Supplementary Table 1, https://stacks.cdc.gov/view/cdc/109086). Among Hispanic persons, Puerto Rican persons had a higher prevalence of asthma (14.0%) than Mexican (5.4%) and other Hispanic persons (6.3%) (Figure 1) (Supplementary Table 1, https://stacks.cdc.gov/view/cdc/109086).

Current asthma prevalence differed by poverty level. Current asthma was more prevalent among persons with family incomes <100% of the FPL (11.4%) and among persons with family incomes 100% to <250% of the FPL (8.3%) than among persons with family incomes at or above 450% of the FPL (6.8%) (Figure 1) (Supplementary Table 1, https://stacks.cdc.gov/view/cdc/109086).

#### Prevalence of Current Asthma by Geographic Location 

Overall, the current asthma prevalence was higher in the Northeast (8.9%) than in the South (7.6%) and the West (7.7%); among adults, the current asthma prevalence was higher in the Northeast (8.8%) than in the South (7.4%) (Table 1). Among children, the prevalence was higher in the Northeast (9.1%) than in the West (6.9%) (Table 1).

**FIGURE 3:**
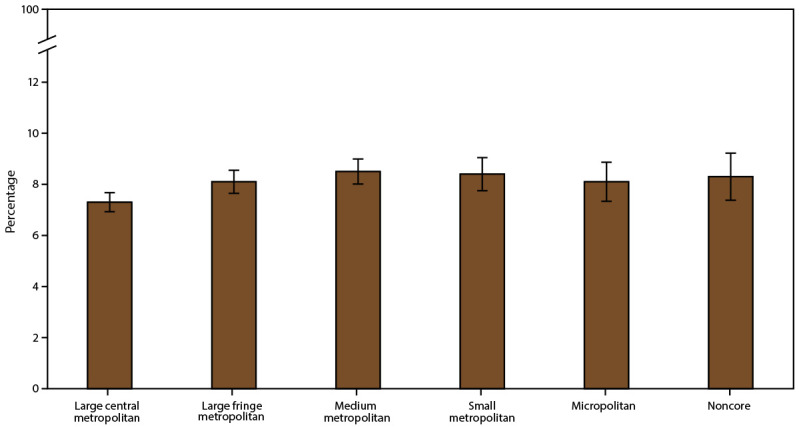
Current asthma* prevalence,^†^ by urban-rural classification^§^ — United States, 2016–2018 **Source:** CDC, National Center for Health Statistics, National Health Interview Survey. https://www.cdc.gov/nchs/nhis/index.htm **Abbreviation:** MSA = metropolitan statistical area. * Includes persons who answered “yes” to the questions, “Have you ever been told by a doctor or other health professional that you had asthma?” and “Do you still have asthma?” ^†^ Prevalence is the proportion of the population who reported having current asthma, with 95% confidence intervals indicated by error bars. ^§^ Large central metropolitan areas are counties in MSAs of ≥1 million population containing the principal city; large fringe metropolitan areas are counties in MSAs of ≥1 million population not containing the principal city; medium metropolitan areas are counties in MSAs of 250,000–999,999 population; small metropolitan areas are counties in MSAs of <250,000 population; micropolitan areas have an urban cluster population of 10,000–49,999; and noncore areas include nonmetropolitan areas that did not qualify as micropolitan, including those without an urban cluster population of at least 10,000.

The prevalence of current asthma overall and among adults was higher in small MSAs (all ages: 8.4%; adults: 8.3%) than in large MSAs (all ages: 7.7%; adults: 7.7%) (Table 1). Current asthma differed among U.S. Census regions for small MSAs. In the small MSA areas, asthma prevalence was higher in the Northeast (10.2%) than in the South (7.8%) (Table 2). Among urban-rural classification categories, the prevalence was higher in the medium metropolitan areas (8.5%) and small metropolitan areas (8.4%) than in large central metropolitan areas (7.3%) (Figure 3) (Table 1).

**TABLE 2:** Prevalence of current asthma* among all ages, children aged 0–17 years, and adults aged ≥18 years, by U.S. Census region and metropolitan statistical area category^†^ — United States, 2016–2018

U.S. Census region	Current asthma								
	Large MSA			Small MSA			Non-MSA		
	Weighted no.^§^	Prevalence % (SE)	(95% CI)	Weighted no.^§^	Prevalence % (SE)	(95% CI)	Weighted no.^§^	Prevalence % (SE)	(95% CI)
**All ages**	**p = 0.03^¶^**			**p = 0.01^¶^**			**p = 0.34**		
Northeast	3,143,580	8.3 (0.34)	(7.6–9.0)	1,496,626	10.2 (0.62)	(9.0–11.4)	410,230	9.8 (1.06)	(7.9–12.1)
Midwest	2,819,643	8.1 (0.30)	(7.5–8.7)	1,638,536	8.2 (0.39)	(7.5–9.0)	1,211,563	8.1 (0.53)	(7.1–9.2)
South	4,710,141	7.5 (0.25)	(7.0–8.0)	2,853,692	7.8 (0.29)	(7.2–8.4)	1,338,634	7.8 (0.47)	(6.9–8.8)
West	3,308,392	7.1 (0.30)	(6.6–7.8)	2,093,839	8.5 (0.41)	(7.7–9.3)	461,591	8.4 (0.70)	(7.1–9.8)
**Children**	p = 0.07			p = 0.03^¶^			p = 0.84		
Northeast	722,244	8.6 (0.78)	(7.2–10.2)	352,346	10.9 (1.26)	(8.7–13.7)	67,505	7.5 (1.54)	(4.9–11.1)
Midwest	609,728	7.8 (0.57)	(6.8–9.0)	346,177	7.7 (0.60)	(6.6–9.0)	287,892	8.9 (1.03)	(7.1–11.1)
South	1,212,476	8.0 (0.48)	(7.1–9.0)	802,969	9.4 (0.64)	(8.2–10.7)	309,314	8.2 (0.87)	(6.6–10.1)
West	697,884	6.6 (0.48)	(5.7–7.6)	447,396	7.4 (0.68)	(6.2–8.9)	92,007	7.4 (1.90)	(4.4–12.1)
**Adults**	p = 0.06			p = 0.001^¶^			p = 0.19		
Northeast	2,421,336	8.2 (0.39)	(7.5–9.0)	1,144,280	9.9 (0.66)	(8.7–11.3)	342,725	10.4 (1.32)	(8.1–13.3)
Midwest	2,209,915	8.2 (0.34)	(7.5–8.9)	1,292,359	8.4 (0.46)	(7.5–9.3)	923,670	7.8 (0.50)	(6.9–8.9)
South	3,497,666	7.3 (0.26)	(6.8–7.8)	2,050,723	7.3 (0.34)	(6.7–8.0)	1,029,320	7.7 (0.50)	(6.7–8.7)
West	2,610,508	7.3 (0.33)	(6.7–8.0)	1,646,443	8.8 (0.41)	(8.1–9.7)	369,583	8.6 (0.71)	(7.4–10.1)

**Source:** CDC, National Center for Health Statistics, National Health Interview Survey. https://www.cdc.gov/nchs/nhis/index.htm

**Abbreviations:** CI = confidence interval; MSA = metropolitan statistical area; SE = standard error.

* Includes persons who answered “yes” to the questions, “Have you ever been told by a doctor or other health professional that you had asthma?” and “Do you still have asthma?”

^†^ Large MSAs have a population of ≥1 million; small MSAs have a population of <1 million. Non-MSAs consist of persons not living in an MSA.

^§^ National Health Interview Survey sample weights were used to adjust for nonresponse, poststratification, and probability of selection to provide estimates for the intended U.S. populations.

^¶^ Statistically significant by chi-square test, p<0.05.

The prevalence of persons with current asthma varied by state. The median percentage was 8.3%, ranging from 5.0% in Nebraska to 12.3% in Maine. The prevalence differed by MSA category in California, Colorado, Mississippi, Oklahoma, and Tennessee but not by the same patterns in each state (Table 3). In California, asthma prevalence was higher in non-MSAs (11.2%) than in large MSAs (6.4%), whereas in Colorado, the prevalence was higher in small MSAs (10.9%) than in non-MSAs (6.6%). In Mississippi, the prevalence was higher in small MSAs (9.3%) than in large MSAs (3.2%) and non-MSAs (5.0%), and in Oklahoma, the prevalence was higher in large MSAs (10.6%) than in small MSAs (5.3%). In Tennessee, the prevalence was higher in small MSAs (8.7%) and in non-MSAs (8.3%) than in large MSAs (4.5%).

**TABLE 3:** Prevalence of current asthma,* by state/area and metropolitan statistical area category^†^ — United States, 2016–2018

State/Area	Current asthma								
	Total		Large MSA		Small MSA		Non-MSA		p value
	Prevalence % (SE)	(95% CI)	Prevalence % (SE)	(95% CI)	Prevalence % (SE)	(95% CI)	Prevalence % (SE)	(95% CI)	
Alabama	**8.5 (0.85)**	**(7.0–10.3)**	11.2 (2.07)	(7.7–15.9)	8.3 (0.81)	(6.8–10.0)	6.3 (1.45)	(4.0–9.8)	0.24
Alaska	**8.4 (0.78)**	**(7.0–10.0)**	—^§^	—^§^	8.8 (0.95)	(7.1–10.8)	7.5 (0.00)	(7.5–7.5)	0.29
Arizona	**8.6 (0.77)**	**(7.2–10.2)**	8.5 (1.02)	(6.7–10.7)	8.8 (0.67)	(7.6–10.2)	—^§^	—^§^	0.78
Arkansas	**8.4 (0.94)**	**(6.7–10.4)**	—^§^	—^§^	9.3 (1.19)	(7.2–11.9)	6.2 (1.62)	(3.7–10.3)	0.13
California	**7.0 (0.32)**	**(6.4–7.7)**	6.4 (0.38)	(5.7–7.2)	8.9 (0.72)	(7.6–10.4)	11.2 (0.00)	(11.2–11.2)	0.001^¶^
Colorado	**9.1 (0.96)**	**(7.4–11.2)**	8.0 (1.59)	(5.4–11.8)	10.9 (0.61)	(9.8–12.2)	6.6 (0.00)	(6.6–6.6)	0.03^¶^
Connecticut	**11.2 (0.92)**	**(9.5–13.2)**	11.9 (2.17)	(8.3–16.9)	10.7 (0.91)	(9.0–12.6)	13.1 (1.60)	(10.3–16.6)	0.37
Delaware	**10.3 (1.42)**	**(7.9–13.5)**	8.3 (1.74)	(5.4–12.4)	12.9 (1.99)	(9.5–17.3)	—^§^	—^§^	0.10
District of Columbia	**12.0 (1.43)**	**(9.5–15.1)**	12.0 (1.43)	(9.5–15.1)	—^§^	—^§^	—^§^	—^§^	—^§^
Florida	**7.1 (0.47)**	**(6.3–8.1)**	7.5 (0.58)	(6.5–8.8)	6.1 (0.79)	(4.7–7.8)	10.1 (0.00)	(10.1–10.1)	0.17
Georgia	**7.9 (0.57)**	**(6.9–9.1)**	7.3 (0.82)	(5.9–9.1)	9.3 (0.45)	(8.4–10.2)	8.4 (1.49)	(5.9–11.9)	0.16
Hawaii	**5.8 (0.66)**	**(4.6–7.2)**	—^§^	—^§^	5.8 (0.80)	(4.4–7.6)	5.8 (0.11)	(5.6–6.0)	0.97
Idaho	**7.4 (0.59)**	**(6.3–8.7)**	—^§^	—^§^	7.9 (0.72)	(6.6–9.4)	6.5 (0.44)	(5.7–7.4)	0.20
Illinois	**7.4 (0.44)**	**(6.6–8.3)**	6.8 (0.53)	(5.9–7.9)	9.4 (0.77)	(7.9–11.0)	8.4 (1.94)	(5.3–13.1)	0.06
Indiana	**8.6 (0.59)**	**(7.5–9.8)**	9.3 (0.87)	(7.7–11.1)	8.0 (0.91)	(6.3–9.9)	7.7 (0.61)	(6.6–9.0)	0.34
Iowa	**8.1 (0.98)**	**(6.4–10.3)**	—^§^	—^§^	7.3 (1.04)	(5.5–9.6)	9.7 (1.65)	(6.9–13.5)	0.20
Kansas	**8.4 (0.53)**	**(7.5–9.5)**	8.5 (1.04)	(6.7–10.8)	9.1 (1.13)	(7.2–11.6)	7.8 (0.41)	(7.0–8.7)	0.31
Kentucky	**8.0 (0.80)**	**(6.5–9.7)**	8.2 (0.66)	(7.0–9.6)	7.3 (1.10)	(5.4–9.8)	7.9 (1.70)	(5.1–11.9)	0.84
Louisiana	**8.2 (0.83)**	**(6.7–10.0)**	6.3 (1.68)	(3.7–10.6)	8.1 (0.95)	(6.4–10.2)	11.7 (1.10)	(9.7–14.0)	0.14
Maine	**12.3 (1.73)**	**(9.3–16.2)**	—^§^	—^§^	14.7 (2.77)	(10.0–21.0)	10.3 (1.32)	(8.0–13.2)	0.23
Maryland	**8.9 (1.15)**	**(6.9–11.4)**	8.9 (1.15)	(6.9–11.4)	—^§^	—^§^	—^§^	—^§^	—^§^
Massachusetts	**10.4 (1.03)**	**(8.6–12.6)**	9.8 (1.07)	(7.9–12.1)	12.1 (2.46)	(8.0–17.8)	10.2 (1.87)	(7.1–14.5)	0.70
Michigan	**8.9 (0.52)**	**(7.9–10.0)**	10.2 (0.79)	(8.8–11.9)	7.3 (0.90)	(5.7–9.2)	8.7 (0.43)	(7.9–9.6)	0.06
Minnesota	**7.2 (0.65)**	**(6.0–8.6)**	7.2 (0.93)	(5.6–9.3)	7.6 (0.00)	(7.6–7.6)	6.9 (1.09)	(5.0–9.3)	0.77
Mississippi	**7.5 (0.97)**	**(5.8–9.6)**	3.2 (0.00)	(3.2–3.2)	9.3 (0.56)	(8.3–10.5)	5.0 (0.79)	(3.7–6.8)	0.001**^¶^**
Missouri	**8.8 (0.86)**	**(7.3–10.7)**	8.6 (1.10)	(6.6–11.0)	9.5 (0.88)	(7.9–11.4)	9.0 (2.27)	(5.5–14.6)	0.82
Montana	**8.3 (0.69)**	**(7.1–9.8)**	—^§^	—^§^	7.0 (0.01)	(7.0–7.0)	8.9 (0.76)	(7.5–10.5)	0.17
Nebraska	**5.0 (0.57)**	**(4.0–6.2)**	—^§^	—^§^	5.0 (0.67)	(3.8–6.5)	4.9 (1.00)	(3.3–7.3)	0.93
Nevada	**8.7 (0.86)**	**(7.1–10.5)**	8.9 (1.14)	(6.9–11.5)	8.1 (0.09)	(7.9–8.3)	6.7 (0.00)	(6.7–6.7)	0.56
New Hampshire	**10.9 (1.00)**	**(9.1–13.1)**	10.4 (0.91)	(8.7–12.3)	9.9 (1.94)	(6.7–14.4)	12.1 (1.54)	(9.4–15.5)	0.72
New Jersey	**7.4 (0.65)**	**(6.2–8.8)**	7.1 (0.74)	(5.8–8.7)	8.8 (0.96)	(7.1–10.9)	—^§^	—^§^	0.18
New Mexico	**9.4 (1.23)**	**(7.2–12.1)**	—^§^	—^§^	9.8 (1.91)	(6.6–14.2)	8.5 (0.57)	(7.5–9.7)	0.59
New York	**8.5 (0.49)**	**(7.6–9.5)**	8.1 (0.55)	(7.1–9.2)	10.0 (1.38)	(7.6–13.1)	10.8 (1.98)	(7.5–15.3)	0.17
North Carolina	**7.5 (0.59)**	**(6.4–8.7)**	6.6 (0.62)	(5.5–8.0)	8.4 (1.04)	(6.6–10.7)	6.5 (0.80)	(5.1–8.3)	0.28
North Dakota	**6.3 (0.97)**	**(4.6–8.5)**	—^§^	—^§^	6.6 (0.00)	(6.6–6.6)	6.1 (1.52)	(3.7–9.9)	0.76
Ohio	**9.0 (0.69)**	**(7.7–10.4)**	8.3 (0.77)	(6.9–9.9)	10.2 (1.40)	(7.7–13.3)	9.2 (2.24)	(5.6–14.6)	0.47
Oklahoma	**9.1 (1.13)**	**(7.2–11.6)**	10.6 (1.20)	(8.5–13.2)	5.3 (1.26)	(3.3–8.4)	10.5 (2.79)	(6.2–17.4)	0.009**^¶^**
Oregon	**8.8 (1.13)**	**(6.8–11.3)**	7.8 (1.62)	(5.1–11.6)	8.6 (1.18)	(6.6–11.2)	13.9 (0.00)	(13.9–13.9)	0.51
Pennsylvania	**8.3 (0.66)**	**(7.1–9.7)**	8.0 (0.73)	(6.6–9.5)	9.3 (1.19)	(7.2–11.9)	5.6 (0.76)	(4.3–7.3)	0.20
Rhode Island	**9.6 (0.78)**	**(8.7–11.2)**	9.6 (0.78)	(8.1–11.2)	—^§^	—^§^	—^§^	—^§^	—^§^
South Carolina	**7.8 (0.89)**	**(6.2–9.7)**	—^§^	—^§^	7.4 (0.93)	(5.8–9.5)	9.9 (2.34)	(6.1–15.5)	0.37
South Dakota	**6.0 (1.17)**	**(4.1–8.8)**	—^§^	—^§^	5.7 (1.58)	(3.3–9.7)	6.7 (0.00)	(6.7–6.7)	0.58
Tennessee	**6.5 (0.49)**	**(5.6–7.5)**	4.5 (0.67)	(3.4–6.0)	8.7 (1.32)	(6.4–11.6)	8.3 (0.43)	(7.5–9.2)	<0.001**^¶^**
Texas	**6.8 (0.37)**	**(6.1–7.6)**	6.8 (0.44)	(6.0–7.8)	7.2 (0.89)	(5.6–9.1)	6.0 (0.61)	(4.9–7.3)	0.48
Utah	**7.3 (1.46)**	**(4.9–10.8)**	8.0 (1.51)	(5.5–11.5)	6.9 (2.26)**	(3.6–12.9)	—^††^	—^††^	—^††^
Vermont	**11.0 (1.67)**	**(8.1–14.7)**	—^§^	—^§^	9.5 (0.98)	(7.7–11.6)	14.5 (0.00)	(14.5–14.5)	0.09
Virginia	**8.0 (0.63)**	**(6.8–9.3)**	8.0 (0.57)	(7.0–9.2)	5.6 (0.76)	(4.2–7.3)	9.5 (2.25)	(5.9–14.9)	0.24
Washington	**7.7 (0.52)**	**(6.8–8.8)**	8.7 (0.64)	(7.5–10.0)	6.6 (1.04)	(4.8–9.0)	7.0 (0.64)	(5.8–8.3)	0.11
West Virginia	**9.1 (0.81)**	**(7.6–10.8)**	—^§^	—^§^	9.6 (0.40)	(8.9–10.5)	7.8 (1.64)	(5.1–11.7)	0.44
Wisconsin	**7.7 (0.63)**	**(6.5–9.0)**	7.5 (0.78)	(6.1–9.1)	9.3 (0.48)	(8.4–10.3)	7.2 (1.33)	(5.0–10.3)	0.19
Wyoming	**8.2 (1.50)**	**(5.7–11.7)**	—^§^	—^§^	8.7 (0.00)	(8.7–8.7)	8.0 (2.07)	(4.8–13.1)	0.75

**Source:** CDC, National Center for Health Statistics, National Health Interview Survey. https://www.cdc.gov/nchs/nhis/index.htm

**Abbreviations:** CI = confidence interval; MSA = metropolitan statistical area; SE = standard error.

* Includes persons who answered “yes” to the questions, “Have you ever been told by a doctor or other health professional that you had asthma?” and “Do you still have asthma?”

^†^ Large MSAs have a population of ≥1 million; small MSAs have a population of <1 million. Non-MSAs consist of persons not living in an MSA.

^§^ State/area does not have the corresponding MSA category.

^¶^ Statistically significant by chi-square test, p<0.05.

** Relative SE = 30%–50%; estimate is unreliable.

^††^ Suppressed because relative SE ≥50%.

### Prevalence of Asthma Attacks in the Past 12 Months Among Persons with Current Asthma

During 2016–2018, approximately 46.0% of the U.S. population with current asthma reported having had one or more asthma attacks in the past 12 months. The prevalence was higher among children aged 0–17 years (53.0%) than adults aged ≥18 years (43.9%) (Table 4).

**TABLE 4:** Asthma attack* prevalence in the past 12 months among all ages, children aged 0–17 years, and adults aged ≥18 years with current asthma,^†^ by geographic area — United States, 2016–2018

Characteristic	Asthma attack								
	All ages			Children			Adults		
	Weighted no.^§^	Prevalence % (SE)	(95% CI)	Weighted no.^§^	Prevalence % (SE)	(95% CI)	Weighted no.^§^	Prevalence % (SE)	(95% CI)
**Total**	**11,703,647**	**46.0 (0.69)**	**(44.6–47.4)**	**3,146,587**	**53.0 (1.33)**	**(50.4–55.6)**	**8,557,060**	**43.9 (0.77)**	**(42.4–45.4)**
**U.S. Census region**	p = 0.28			p = 0.16			p = 0.20		
Northeast	2,214,856	43.9 (1.47)	(41.0–46.8)	619,571	54.2 (2.90)	(48.5–59.9)	1,595,285	40.8 (1.61)	(37.7–44.0)
Midwest	2,557,512	45.2 (1.41)	(42.4–48.0)	624,634	50.5 (2.92)	(44.8–56.2)	1,932,878	43.7 (1.56)	(40.7–46.8)
South	4,156,819	46.8 (1.27)	(44.3–49.3)	1,183,284	50.9 (2.17)	(46.7–55.2)	2,973,535	45.3 (1.45)	(42.5–48.2)
West	2,774,459	47.4 (1.38)	(44.7–50.1)	719,097	58.3 (2.91)	(52.5–63.9)	2,055,362	44.5 (1.43)	(41.7–47.3)
**MSA category^¶^**	p = 0.53			p = 0.09			p = 0.05		
Large MSA	6,321,586	45.3 (1.00)	(43.3–47.3)	1,797,752	55.7 (1.90)	(51.9–59.3)	4,523,834	42.2 (1.10)	(40.0–44.3)
Small MSA	3,771,354	46.7 (1.20)	(44.4–49.1)	968,333	49.7 (2.06)	(45.7–53.7)	2,803,022	45.8 (1.37)	(43.1–48.5)
Non-MSA	1,610,707	47.1 (1.69)	(43.8–50.4)	380,502	50.3 (3.43)	(43.6–57.0)	1,230,205	46.2 (1.86)	(42.6–49.8)
Urban–rural classification**	p = 0.80			p = 0.28			p = 0.17		
Large central metropolitan	3,308,972	45.2 (1.32)	(42.6–47.8)	928,878	54.8 (2.64)	(49.6–59.9)	2,380,094	42.3 (1.44)	(39.5–45.2)
Large fringe metropolitan	3,051,464	45.6 (1.46)	(42.8–48.5)	873,512	56.7 (2.77)	(51.2–62.0)	2,177,951	42.3 (1.58)	(39.2–45.4)
Medium metropolitan	2,538,772	45.8 (1.49)	(42.9–48.7)	687,147	49.7 (2.49)	(44.9–54.6)	1,851,625	44.5 (1.74)	(41.1–47.9)
Small metropolitan	1,193,733	48.3 (1.97)	(44.4–52.2)	276,547	49.2 (3.81)	(41.8–56.6)	917,186	48.0 (2.13)	(43.9–52.2)
Micropolitan	1,014,584	47.7 (1.98)	(43.8–51.6)	225,660	47.9 (4.06)	(40.1–55.9)	788,924	47.6 (2.33)	(43.1–52.2)
Noncore	596,123	46.1 (2.76)	(40.8–51.5)	154,843	54.2 (6.28)	(41.9–66.1)	441,280	43.8 (2.93)	(38.1–49.6)

**Source:** CDC, National Center for Health Statistics, National Health Interview Survey. https://www.cdc.gov/nchs/nhis/index.htm

**Abbreviations:** CI = confidence interval; MSA = metropolitan statistical area; SE = standard error.

* Having had one or more episodes of asthma or an asthma attack in the past 12 months.

^†^ Includes persons who answered “yes” to the questions, “Have you ever been told by a doctor or other health professional that you had asthma?” and “Do you still have asthma?”

^§^ National Health Interview Survey sample weights were used to adjust for nonresponse, poststratification, and probability of selection to provide estimates for the intended U.S. populations.

^¶^ Large MSAs have a population of ≥1 million; small MSAs have a population of <1 million. Non-MSAs consist of persons not living in an MSA.

** Large central metropolitan areas are counties in MSAs of ≥1 million population containing the principal city; large fringe metropolitan areas are counties in MSAs of ≥1 million population not containing the principal city; medium metropolitan areas are counties in MSAs of 250,000–999,999 population; small metropolitan areas are counties in MSAs of <250,000 population; micropolitan areas have an urban cluster population of 10,000–49,999; and noncore areas include nonmetropolitan areas that did not qualify as micropolitan, including those without an urban cluster population of at least 10,000.

#### Trends in Prevalence of Asthma Attacks 

**FIGURE 4:**
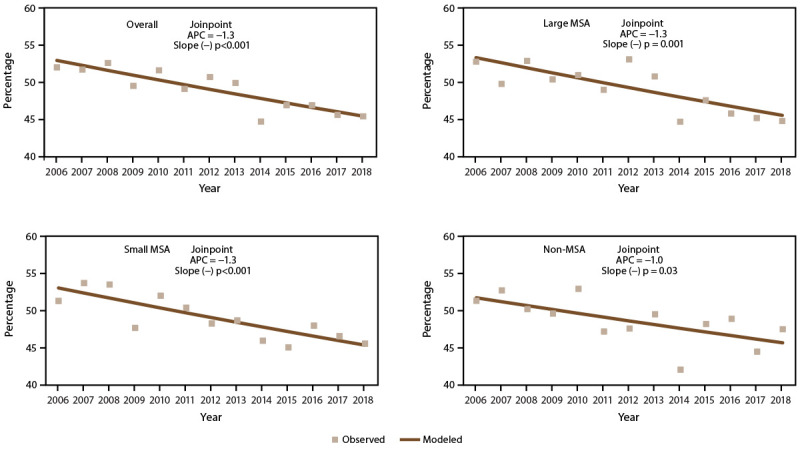
Asthma attack prevalence* among persons with current asthma,^†^ by year and metropolitan statistical area category^§^ — United States, 2006–2018 **Source:** CDC, National Center for Health Statistics, National Health Interview Survey. https://www.cdc.gov/nchs/nhis/index.htm **Abbreviations:** APC = annual percent change; MSA = metropolitan statistical area. * Prevalence is the proportion of the population with current asthma who reported having one or more episodes of asthma or an asthma attack in the past 12 months. Significance set at p<0.05. ^†^ Includes persons who answered “yes” to the questions, “Have you ever been told by a doctor or other health professional that you had asthma?” and “Do you still have asthma?” ^§^ Large MSAs have a population of ≥1 million; small MSAs have a population of <1 million. Non-MSAs consist of persons not living in an MSA.

The overall asthma attack prevalence and prevalence for each MSA category decreased significantly during 2006–2018 (Figure 4) (Supplementary Tables 9 and 23, https://stacks.cdc.gov/view/cdc/109086). Although decreases in asthma attack prevalence were observed among children and adults with current asthma, the overall decreasing trend was primarily observed among adults (not considering MSA categories) (APC = −1.3), among adults in large MSAs (APC = −1.7), and in small MSAs (APC = −1.0). Among children, a decreasing trend was only significant in small MSAs (APC = −1.8) (Supplementary Table 23, https://stacks.cdc.gov/view/cdc/109086).

#### Prevalence of Asthma Attacks by Demographic Characteristics and Poverty Level

**FIGURE 5:**
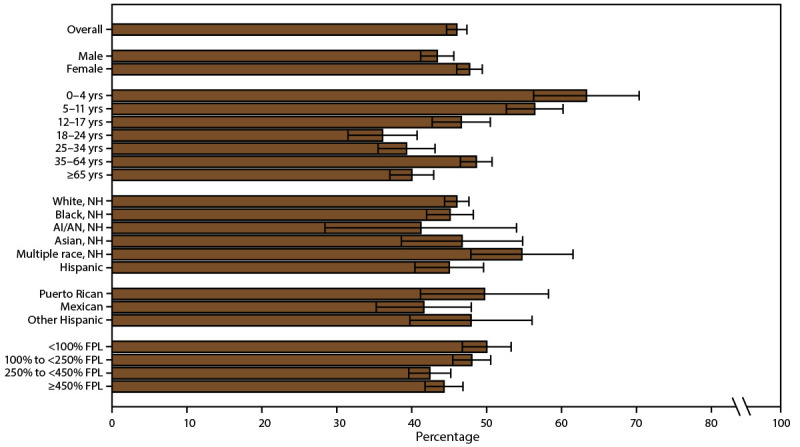
Asthma attack prevalence* among persons with current asthma,^†^ by sex, age group, race/ethnicity,^§^ and federal poverty level^¶^ — United States, 2016–2018 **Source:** CDC, National Center for Health Statistics, National Health Interview Survey. https://www.cdc.gov/nchs/nhis/index.htm **Abbreviations:** AI/AN = American Indian or Alaska Native; FPL = federal poverty level; NH = non-Hispanic. * Prevalence is the proportion of the population with current asthma who reported having one or more episodes of asthma or an asthma attack in the past 12 months, with 95% confidence intervals indicated by error bars. ^†^ Includes persons who answered “yes” to the questions, “Have you ever been told by a doctor or other health professional that you had asthma?” and “Do you still have asthma?” ^§^ Puerto Rican, Mexican, and other Hispanic are subsets of Hispanic. ^¶^ Determined by family income and size using U.S. Census Bureau poverty thresholds. Poverty level is defined as the ratio of family income to federal poverty threshold in terms of FPL.

Asthma attack prevalence in the past 12 months differed by demographic characteristics (Figure 5) (Supplementary Table 7, https://stacks.cdc.gov/view/cdc/109086) and was higher among females (47.7%) than males (43.4%) (Figure 5) (Supplementary Table 7, https://stacks.cdc.gov/view/cdc/109086). Asthma attack prevalence was highest among children aged 0–4 years (63.3%) and adults aged 35–64 years (48.6%) (Figure 5) (Supplementary Table 7, https://stacks.cdc.gov/view/cdc/109086). Multiple-race persons had a higher prevalence of asthma attacks (54.7%) than White persons (46.0%). No other significant differences in asthma attacks were observed by race or ethnicity (Figure 5) (Supplementary Table 7, https://stacks.cdc.gov/view/cdc/109086).

Asthma attack prevalence differed by poverty level. Asthma attacks were more prevalent among persons with family incomes <100% of the FPL (50.0%) and among persons with family incomes 100% to <250% of the FPL (48.0%) compared with persons with family incomes 250% to <450% of the FPL (42.4%) (Figure 5) (Supplementary Table 7, https://stacks.cdc.gov/view/cdc/109086).

#### Prevalence of Asthma Attacks by Geographic Location 

**FIGURE 6:**
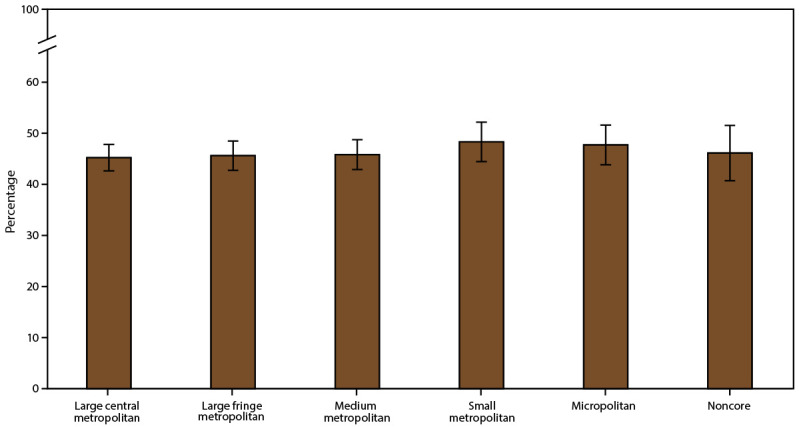
Asthma attack prevalence* among persons with current asthma,^†^ by urban-rural classification^§^ — United States, 2016–2018 **Source:** CDC, National Center for Health Statistics, National Health Interview Survey. https://www.cdc.gov/nchs/nhis/index.htm **Abbreviation:** MSA = metropolitan statistical area. * Prevalence is the proportion of the population with current asthma who reported having one or more episodes of asthma or an asthma attack in the past 12 months, with 95% confidence intervals indicated by error bars. ^†^ Includes persons who answered “yes” to the questions, “Have you ever been told by a doctor or other health professional that you had asthma?” and “Do you still have asthma?” ^§^ Large central metropolitan areas are counties in MSAs of ≥1 million population containing the principal city; large fringe metropolitan areas are counties in MSAs of ≥1 million population not containing the principal city; medium metropolitan areas are counties in MSAs of 250,000–999,999 population; small metropolitan areas are counties in MSAs of <250,000 population; micropolitan areas have an urban cluster population of 10,000–49,999; and noncore areas include nonmetropolitan areas that did not qualify as micropolitan, including those without an urban cluster population of at least 10,000.

Asthma attack prevalence did not differ by U.S. Census region, MSA category, or urban-rural classification (Table 4) (Figure 6). Asthma attack prevalence by MSA category did not differ among U.S. Census regions (Table 5).

**TABLE 5:** Asthma attack* prevalence in the past 12 months among all ages, children aged 0–17 years, and adults aged ≥18 years with current asthma,^†^ by U.S. Census region and metropolitan statistical area category^§^ — United States, 2016–2018

U.S. Census region	Asthma attack								
	Large MSA			Small MSA			Non-MSA		
	Weighted no.^¶^	Prevalence % (SE)	(95% CI)	Weighted no.^¶^	Prevalence % (SE)	(95% CI)	Weighted no.^¶^	Prevalence % (SE)	(95% CI)
**All ages**	**p = 0.07**			**p = 0.08**			**p = 0.11**		
Northeast	1,412,467	44.9 (2.09)	(40.9–49.1)	600,646	40.1 (3.02)	(34.4–46.2)	201,743	49.2 (2.70)	(43.9–54.5)
Midwest	1,144,694	40.7 (1.98)	(36.9–44.6)	816,369	49.9 (3.13)	(43.8–56.0)	596,449	49.3 (2.35)	(44.7–53.9)
South	2,219,116	47.2 (1.75)	(43.8–50.7)	1,377,224	48.4 (2.01)	(44.4–52.3)	560,479	41.9 (3.17)	(35.8–48.2)
West	1,545,308	46.8 (2.18)	(42.5–51.1)	977,114	46.8 (1.78)	(43.3–50.3)	252,037	54.6 (3.99)	(46.7–62.2)
**Children**	p = 0.12			p = 0.35			p = 0.12		
Northeast	427,907	59.2 (4.25)	(50.7–67.2)	148,248	42.1 (4.93)	(32.8–51.9)	43,416	64.3 (6.32)	(51.2–75.6)
Midwest	289,469	48.0 (4.40)	(39.5–56.6)	179,598	51.9 (4.71)	(42.7–61.0)	155,568	54.1 (5.84)	(42.7–65.2)
South	654,941	54.1 (2.97)	(48.2–59.8)	399,287	49.7 (3.23)	(43.4–56.0)	129,057	41.7 (5.14)	(32.1–52.0)
West	425,436	61.3 (4.03)	(53.2–68.9)	241,200	53.9 (3.89)	(46.2–61.4)	52,462	57.0 (6.69)	(43.7–69.4)
**Adults**	p = 0.17			p = 0.16			p = 0.27		
Northeast	984,561	40.7 (2.38)	(36.1–45.4)	452,398	39.5 (3.67)	(32.6–46.9)	158,327	46.2 (3.33)	(39.8–52.8)
Midwest	855,226	38.7 (2.09)	(34.7–42.9)	636,771	49.4 (3.37)	(42.8–55.9)	440,881	47.8 (2.46)	(43.0–52.6)
South	1,564,175	44.9 (2.00)	(41.0–48.8)	977,938	47.8 (2.34)	(43.3–52.4)	431,422	41.9 (3.62)	(35.0–49.2)
West	1,119,873	42.9 (2.29)	(38.5–47.4)	735,915	44.8 (2.01)	(40.9–48.8)	199,575	54.0 (4.77)	(44.6–63.1)

**Source:** CDC, National Center for Health Statistics, National Health Interview Survey. https://www.cdc.gov/nchs/nhis/index.htm

**Abbreviations:** CI = confidence interval; MSA = metropolitan statistical area; SE = standard error.

* Having had one or more episodes of asthma or an asthma attack in the past 12 months.

^†^ Includes persons who answered “yes” to the questions, “Have you ever been told by a doctor or other health professional that you had asthma?” and “Do you still have asthma?”

**^§^** Large MSAs have a population of ≥1 million; small MSAs have a population of <1 million. Non-MSAs consist of persons not living in an MSA.

^¶^ National Health Interview Survey sample weights were used to adjust for nonresponse, poststratification, and probability of selection to provide estimates for the intended U.S. populations.

Asthma attack prevalence varied by state. The median percentage was 47.1%, ranging from 33.8% in Connecticut to 60.9% in Wyoming. Asthma attack prevalence differed by MSA category in Ohio, New York, North Dakota, and Tennessee, although the patterns differed among states (Table 6). In Ohio, the prevalence was highest in small MSAs (49.2%) and non-MSAs (51.8%) compared with large MSAs (33.7%). In New York, the prevalence was highest in non-MSAs (58.7%), followed by large MSAs (46.2%) and small MSAs (29.9%). In North Dakota, the prevalence was higher in small MSAs (49.0%) than in non-MSAs (32.4%); the prevalence estimate for large MSAs was too unreliable to report. In Tennessee, the prevalence was higher in large MSAs (66.5%) than in small MSAs (33.8%) and non-MSAs (40.4%).

**TABLE 6:** Asthma attack* prevalence in the past 12 months among persons with current asthma,^†^ by state/area and metropolitan statistical area category^§^ — United States, 2016–2018

State/Area	Asthma attack								
	Total		Large MSA		Small MSA		Non-MSA		p value
	Prevalence % (SE)	(95% CI)	Prevalence % (SE)	(95% CI)	Prevalence % (SE)	(95% CI)	Prevalence % (SE)	(95% CI)	
Alabama	**46.8 (3.62)**	**(39.8–53.9)**	44.6 (7.65)	(30.5–59.7)	49.1 (4.95)	(39.5–58.7)	45.8 (2.96)	(40.0–51.6)	0.83
Alaska	**60.3 (8.00)**	**(44.0–74.5)**	—^¶^	—^¶^	63.5 (9.42)	(44.0–79.5)	51.6 (0.00)	(51.6–51.6)	0.32
Arizona	**44.2 (4.65)**	**(35.4–53.4)**	45.6 (6.07)	(34.1–57.5)	40.5 (5.80)	(29.8–52.2)	—^¶^	—^¶^	0.53
Arkansas	**55.8 (7.32)**	**(41.3–69.3)**	—^¶^	—^¶^	60.6 (7.71)	(44.9–74.3)	38.5 (3.20)	(32.4–44.9)	0.07
California	**46.2 (2.50)**	**(41.4–51.1)**	45.3 (3.28)	(39.0–51.8)	47.3 (3.56)	(40.4–54.3)	59.7 (0.00)	(59.7–59.7)	0.57
Colorado	**44.6 (3.13)**	**(38.5–50.7)**	48.8 (6.53)	(36.3–61.4)	42.6 (3.31)	(36.3–49.2)	34.1 (0.00)	(34.1–34.1)	0.43
Connecticut	**33.8 (5.36)**	**(24.2–45.0)**	43.3 (9.54)	(26.3–62.1)	28.4 (6.05)	(18.1–41.5)	32.0 (14.01)**	(11.8–62.5)	0.49
Delaware	**48.6 (4.32)**	**(40.2–57.0)**	53.0 (5.95)	(41.4–64.3)	45.1 (5.68)	(34.3–56.3)	—^¶^	—^¶^	0.36
District of Columbia	**50.0 (6.25)**	**(38.0–62.0)**	50.0 (6.25)	(38.0–62.0)	—^¶^	—^¶^	—^¶^	—^¶^	—^¶^
Florida	**49.0 (3.03)**	**(43.1–54.9)**	50.7 (3.86)	(43.1–58.2)	47.2 (4.78)	(38.0–56.5)	—^††^	—^††^	0.50
Georgia	**51.3 (4.70)**	**(42.1–60.4)**	49.5 (6.23)	(37.5–61.5)	44.2 (7.96)	(29.7–59.9)	67.0 (6.77)	(52.7–78.8)	0.15
Hawaii	**47.9 (5.86)**	**(36.7–59.3)**	—^¶^	—^¶^	47.3 (7.04)	(34.0–61.0)	50.7 (2.59)	(45.6–55.7)	0.68
Idaho	**52.7 (7.38)**	**(38.4–66.6)**	—^¶^	—^¶^	43.0 (7.43)	(29.4–57.8)	76.3 (7.33)	(59.2–87.7)	0.12
Illinois	**41.3 (4.22)**	**(33.3–49.7)**	37.8 (5.30)	(28.1–48.6)	50.9 (8.36)	(35.0–66.6)	42.6 (5.44)	(32.4–53.5)	0.44
Indiana	**46.1 (2.93)**	**(40.5–51.9)**	40.3 (5.34)	(30.4–51.1)	55.4 (2.56)	(50.4–60.4)	48.7 (3.11)	(42.7–54.8)	0.11
Iowa	**37.0 (4.68)**	**(28.4–46.6)**	—^¶^	—^¶^	33.7 (7.09)	(21.4–48.6)	41.7 (2.55)	(36.8–46.8)	0.32
Kansas	**60.2 (4.28)**	**(51.6–68.2)**	59.9 (13.46)	(33.2–81.8)	62.2 (2.96)	(56.2–67.8)	58.6 (3.54)	(51.5–65.3)	0.77
Kentucky	**39.0 (3.81)**	**(31.9–46.7)**	38.4 (4.29)	(30.4–47.1)	49.4 (10.05)	(30.7–68.2)	35.9 (6.86)	(23.8–50.2)	0.51
Louisiana	**40.6 (6.61)**	**(28.6–54.0)**	36.5 (8.88)	(21.3–54.9)	41.8 (9.89)	(24.4–61.5)	41.7 (12.98)**	(20.1–67.1)	0.90
Maine	**40.3 (5.25)**	**(30.6–50.9)**	—^¶^	—^¶^	41.4 (8.00)	(27.0–57.5)	38.9 (6.36)	(27.4–51.9)	0.81
Maryland	**42.5 (5.00)**	**(33.1–52.4)**	42.5 (5.00)	(33.1–52.4)	—^¶^	—^¶^	—^¶^	—^¶^	—^¶^
Massachusetts	**40.4 (5.55)**	**(30.1–51.6)**	39.5 (5.58)	(29.2–50.8)	41.0 (12.89)**	(19.6–66.4)	52.7 (10.40)	(32.9–71.6)	0.60
Michigan	**48.6 (4.49)**	**(39.9–57.3)**	40.4 (5.33)	(30.5–51.1)	59.5 (10.97)	(37.6–78.2)	55.0 (6.55)	(42.1–67.2)	0.11
Minnesota	**49.6 (5.03)**	**(39.9–59.4)**	48.3 (6.70)	(35.6–61.3)	50.8 (0.00)	(50.8–50.8)	52.3 (10.60)	(32.3–71.6)	0.92
Mississippi	**50.2 (4.07)**	**(42.3–58.1)**	—^††^	—^††^	—^††^	—^††^	42.0 (6.34)	(30.3–54.7)	0.21
Missouri	**51.1 (3.90)**	**(43.5–58.7)**	51.1 (4.24)	(42.9–59.4)	58.8 (15.08)	(29.6–82.9)	45.2 (2.65)	(40.1–50.4)	0.36
Montana	**55.7 (4.48)**	**(46.8–64.2)**	—^¶^	—^¶^	64.1 (3.68)	(56.6–71.0)	53.1 (5.14)	(43.0–62.9)	0.15
Nebraska	**34.3 (7.08)**	**(22.0–49.2)**	—^¶^	—^¶^	38.9 (8.16)	(24.5–55.5)	—^††^	—^††^	0.26
Nevada	**37.2 (4.03)**	**(29.7–45.4)**	38.8 (5.12)	(29.4–49.2)	—^††^	—^††^	—^††^	—^††^	0.44
New Hampshire	**48.2 (4.15)**	**(40.2–56.3)**	44.2 (5.16)	(34.4–54.4)	—^††^	—^††^	—^††^	—^††^	0.26
New Jersey	**37.8 (4.31)**	**(29.8–46.5)**	38.8 (4.86)	(29.8–48.6)	33.5 (9.43)	(18.0–53.6)	—^¶^	—^¶^	0.62
New Mexico	**43.3 (5.78)**	**(32.5–54.8)**	—^¶^	—^¶^	44.8 (7.68)	(30.6–59.9)	39.9 (4.84)	(30.9–49.7)	0.63
New York	**44.8 (3.10)**	**(38.8–51.0)**	46.2 (3.52)	(39.4–53.2)	29.9 (4.35)	(22.1–39.0)	58.7 (1.05)	(56.6–60.7)	<0.001^§§^
North Carolina	**53.3 (3.77)**	**(45.9–60.5)**	42.9 (7.03)	(30.0–56.9)	57.0 (5.32)	(46.4–67.0)	62.5 (7.35)	(47.4–75.5)	0.14
North Dakota	**39.1 (6.16)**	**(27.8–51.6)**	—^¶^	—^¶^	49.0 (0.00)	(49.0–49.0)	32.4 (3.37)	(26.2–39.3)	0.02^§§^
Ohio	**42.1 (3.44)**	**(35.5–48.9)**	33.7 (3.34)	(27.5–40.5)	49.2 (7.59)	(34.8–63.7)	51.8 (7.02)	(38.2–65.1)	0.04^§§^
Oklahoma	**34.2 (7.42)**	**(21.4–49.8)**	37.8 (8.58)	(22.9–55.4)	52.6 (14.00)	(26.9–76.9)	23.0 (11.27)**	(7.9–51.0)	0.35
Oregon	**59.2 (3.91)**	**(51.3–66.6)**	59.3 (7.48)	(44.3–72.8)	56.7 (4.62)	(47.5–65.5)	63.3 (0.00)	(63.3–63.3)	0.53
Pennsylvania	**50.9 (2.59)**	**(45.9–56.0)**	51.5 (4.64)	(42.5–60.5)	50.4 (2.57)	(45.3–55.4)	50.1 (2.18)	(45.8–54.4)	0.97
Rhode Island	**49.8 (7.25)**	**(35.9–63.6)**	49.8 (7.25)	(35.9–63.6)	—^¶^	—^¶^	—^¶^	—^¶^	—^¶^
South Carolina	**38.8 (4.19)**	**(31.0–47.3)**	—^¶^	—^¶^	36.1 (4.28)	(28.2–44.8)	50.4 (12.04)	(28.3–72.3)	0.40
South Dakota	**41.0 (0.30)**	**(40.4–41.6)**	—^¶^	—^¶^	41.1 (0.45)	(40.2–41.9)	41.0 (0.00)	(41.0–41.0)	0.86
Tennessee	**47.1 (4.07)**	**(39.3–55.1)**	66.5 (7.44)	(50.7–70.2)	33.8 (4.55)	(25.5–43.2)	40.4 (6.81)	(28.0–54.1)	0.008^§§^
Texas	**43.9 (3.30)**	**(37.5–50.4)**	44.1 (4.13)	(36.3–52.3)	49.1 (6.06)	(37.5–60.8)	28.4 (5.06)	(19.6–39.2)	0.08
Utah	**47.7 (3.98)**	**(40.0–55.5)**	44.1 (5.24)	(34.2–54.4)	—^††^	—^††^	—^††^	—^††^	0.53
Vermont	**47.2 (7.62)**	**(32.9–62.0)**	—^¶^	—^¶^	52.0 (9.39)	(34.1–69.4)	39.8 (0.00)	(39.8–39.8)	0.28
Virginia	**51.5 (3.73)**	**(44.2–58.8)**	56.0 (3.62)	(48.8–62.9)	40.3 (4.76)	(31.4–49.8)	39.9 (10.06)	(22.6–60.2)	0.11
Washington	**52.4 (3.53)**	**(45.5–59.3)**	54.5 (4.55)	(45.6–63.2)	46.1 (4.71)	(37.0–55.3)	59.9 (17.06)	(27.1–85.8)	0.33
West Virginia	**55.3 (11.98)**	**(32.3–76.2)**	—^¶^	—^¶^	59.4 (15.08)	(30.1–83.3)	44.1 (6.94)	(31.3–57.9)	0.40
Wisconsin	**42.7 (4.27)**	**(34.6–51.2)**	38.6 (5.43)	(28.6–49.6)	33.6 (3.58)	(27.0–41.0)	53.5 (9.63)	(35.0–71.1)	0.18
Wyoming	**60.9 (5.53)**	**(49.7–71.1)**	—^¶^	—^¶^	68.1 (0.00)	(68.1–68.1)	57.9 (5.84)	(46.3–68.8)	0.27

**Source:** CDC, National Center for Health Statistics, National Health Interview Survey. https://www.cdc.gov/nchs/nhis/index.htm

**Abbreviations:** CI = confidence interval; MSA = metropolitan statistical area; SE = standard error.

* Having had one or more episodes of asthma or an asthma attack in the past 12 months.

^†^ Includes persons who answered “yes” to the questions, “Have you EVER been told by a doctor or other health professional that you had asthma?” and “Do you still have asthma?”

**^§^** Large MSAs have a population of ≥1 million; small MSAs have a population of <1 million. Non-MSAs consist of persons not living in an MSA.

^¶^ State/area does not have the corresponding MSA category.

** Relative SE = 30%–50%; estimate is unreliable.

^††^ Suppressed because relative SE ≥50%.

**^§§^** Statistically significant by chi-square test, p<0.05.

### Prevalence of Emergency Department and Urgent Care Center Visits Because of Asthma in the Past 12 Months Among Persons with Current Asthma

During 2016–2018, approximately 11.9% (11.1%–12.7%) of the U.S. population with current asthma reported having one or more ED/UCC visits because of asthma within the past 12 months. Almost twice as many children with asthma reported ED/UCC visits (17.9%) than did adults (10.1%) (Table 7).

**TABLE 7:** Prevalence of emergency department and urgent care center visits* because of asthma in the past 12 months among all ages, children aged 0–17 years, and adults aged ≥18 years with current asthma,^†^ by geographic area — United States, 2016–2018

Characteristic	Emergency department and urgent care center visits								
	All ages			Children			Adults		
	Weighted no.^§^	Prevalence % (SE)	(95% CI)	Weighted no.^§^	Prevalence % (SE)	(95% CI)	Weighted no.^§^	Prevalence % (SE)	(95% CI)
**Total**	**3,029,309**	**11.9 (0.42)**	**(11.1–12.7)**	**1,063,589**	**17.9 (1.02)**	**(16.0–20.0)**	**1,965,720**	**10.1 (0.46)**	**(9.2–11.0)**
**U.S. Census region**	p<0.001^¶^			p = 0.73			p = 0.001^¶^		
Northeast	546,245	10.8 (0.80)	(9.3–12.5)	204,250	17.9 (2.05)	(14.2–22.3)	341,995	8.8 (0.86)	(7.2–10.6)
Midwest	547,697	9.7 (0.76)	(8.3–11.3)	195,903	15.8 (2.15)	(12.0–20.5)	351,793	7.9 (0.75)	(6.6–9.6)
South	1,263,983	14.2 (0.79)	(12.7–15.8)	437,283	18.9 (1.79)	(15.6–22.6)	826,700	12.6 (0.92)	(10.9–14.5)
West	671,384	11.5 (0.94)	(9.7–13.4)	226,153	18.3 (2.17)	(14.4–22.9)	445,231	9.6 (1.00)	(7.8–11.8)
MSA category**	p = 0.001^¶^			p = 0.02^¶^			p = 0.03^¶^		
Large MSA	1,831,355	13.1 (0.69)	(11.8–14.5)	651,142	20.1 (1.51)	(17.3–23.3)	1,180,213	11.0 (0.74)	(9.6–12.5)
Small MSA	900,517	11.1 (0.69)	(9.9–12.6)	320,476	16.4 (1.74)	(13.3–20.1)	580,040	9.5 (0.75)	(8.1–11.0)
Non-MSA	297,437	8.7 (0.93)	(7.0–10.7)	91,970	12.2 (2.15)	(8.5–17.0)	205,467	7.7 (0.93)	(6.1–9.7)
**Urban–rural classification^††^**	p = 0.003^¶^			p = 0.02^¶^			p = 0.06		
Large central metropolitan	1,021,621	14.0 (0.87)	(12.3–15.7)	390,746	23.0 (2.13)	(19.1–27.4)	630,875	11.2 (0.92)	(9.5–13.2)
Large fringe metropolitan	817,672	12.2 (1.08)	(10.2–14.5)	262,450	17.0 (2.21)	(13.1–21.8)	555,222	10.8 (1.16)	(8.7–13.3)
Medium metropolitan	601,975	10.8 (0.79)	(9.4–12.5)	232,263	16.8 (2.12)	(13.0–21.4)	369,712	8.9 (0.83)	(7.4–10.6)
Small metropolitan	290,603	11.8 (1.37)	(9.3–14.7)	86,159	15.3 (3.07)	(10.2–22.3)	204,444	10.7 (1.52)	(8.1–14.1)
Micropolitan	200,120	9.4 (1.14)	(7.4–11.9)	62,933	13.4 (2.68)	(8.9–19.6)	137,187	8.3 (1.26)	(6.1–11.1)
Noncore	97,317	7.5 (1.25)	(5.4–10.4)	29,037	10.2 (2.86)	(5.8–17.3)	68,280	6.8 (1.30)	(4.6–9.8)

**Source:** CDC, National Center for Health Statistics, National Health Interview Survey. https://www.cdc.gov/nchs/nhis/index.htm

**Abbreviations:** CI = confidence interval; MSA = metropolitan statistical area; SE = standard error.

* Having had one or more emergency room or urgent care center visits because of asthma in the past 12 months among persons with current asthma.

^†^ Includes persons who answered “yes” to the questions, “Have you ever been told by a doctor or other health professional that you had asthma?” and “Do you still have asthma?”

**^§^** National Health Interview Survey sample weights were used to adjust for nonresponse, poststratification, and probability of selection to provide estimates for the intended U.S. populations.

^¶^ Statistically significant by chi-square test, p<0.05.

** Large MSAs have a population of ≥1 million; small MSAs have a population of <1 million. Non-MSAs consist of persons not living in an MSA.

^††^ Large central metropolitan areas are counties in MSAs of ≥1 million population containing the principal city; large fringe metropolitan areas are counties in MSAs of ≥1 million population not containing the principal city; medium metropolitan areas are counties in MSAs of 250,000–999,999 population; small metropolitan areas are counties in MSAs of <250,000 population; micropolitan areas have an urban cluster population of 10,000–49,999; and noncore areas include nonmetropolitan areas that did not qualify as micropolitan, including those without an urban cluster population of at least 10,000.

#### Trends in Prevalence of Emergency Department and Urgent Care Center Visits

**FIGURE 7:**
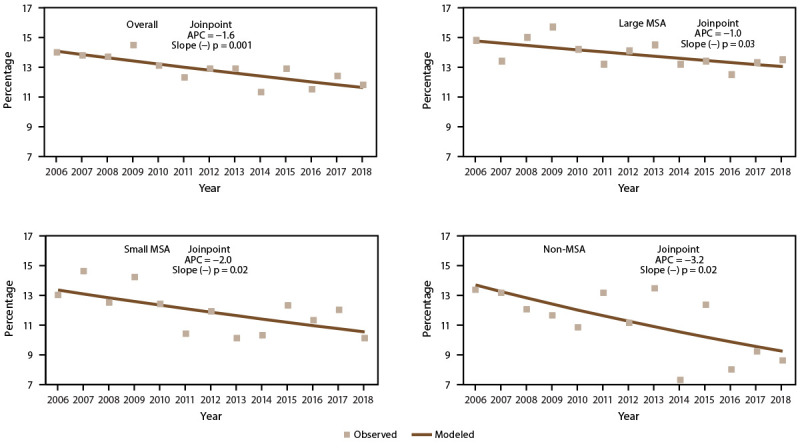
Prevalence of emergency department and urgent care center visits* because of asthma among persons with current asthma,^†^ by year and metropolitan statistical area category^§^ — United States, 2006–2018 **Source:** CDC, National Center for Health Statistics, National Health Interview Survey. https://www.cdc.gov/nchs/nhis/index.htm **Abbreviations:** APC = annual percent change; MSA = metropolitan statistical area. * Prevalence is the proportion of the population with current asthma who reported having had one or more emergency department or urgent care center visits because of asthma in the past 12 months. Significance set at p<0.05. ^†^ Includes persons who answered “yes” to the questions, “Have you ever been told by a doctor or other health professional that you had asthma?” and “Do you still have asthma?” ^§^ Large MSAs have a population of ≥1 million; small MSAs have a population of <1 million. Non-MSAs consist of persons not living in an MSA.

The overall percentage of persons reporting an ED/UCC visit because of asthma decreased during 2006–2018, regardless of MSA category (Figure 7) (Supplementary Tables 15 and 23, https://stacks.cdc.gov/view/cdc/109086). Although decreases in ED/UCC visits were observed among children and adult populations, the trend was significant only among adults (APC = −1.4) (Supplementary Table 23, https://stacks.cdc.gov/view/cdc/109086).

#### Prevalence of Emergency Department and Urgent Care Center Visits by Demographic Characteristics and Poverty Level

**FIGURE 8:**
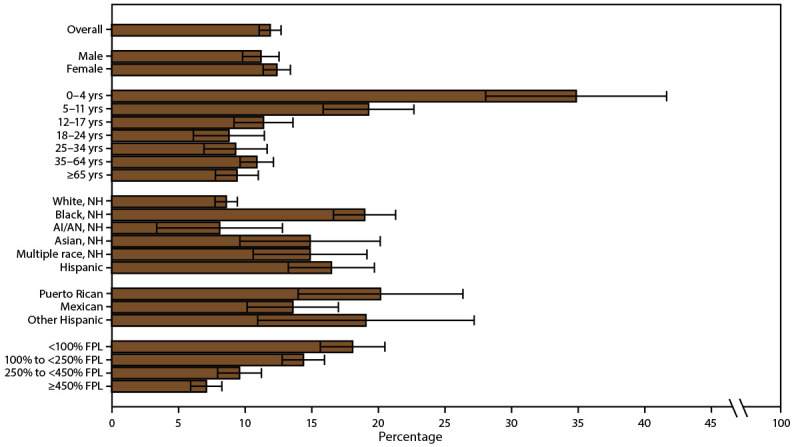
Prevalence of emergency department and urgent care center visits* because of asthma among persons with current asthma,^†^ by sex, age group, race/ethnicity,^§^ and federal poverty level^¶^ — United States, 2016–2018 **Source:** CDC, National Center for Health Statistics, National Health Interview Survey. https://www.cdc.gov/nchs/nhis/index.htm **Abbreviations:** AI/AN = American Indian or Alaska Native; FPL = federal poverty level; NH = non-Hispanic. * Prevalence is the proportion of the population with current asthma who reported having had one or more emergency department or urgent care center visits because of asthma in the past 12 months, with 95% confidence intervals indicated by error bars. ^†^ Includes persons who answered “yes” to the questions, “Have you ever been told by a doctor or other health professional that you had asthma?” and “Do you still have asthma?” ^§^ Puerto Rican, Mexican, and other Hispanic are subsets of Hispanic. ^¶^ Determined by family income and size using U.S. Census Bureau poverty thresholds. Poverty level is defined as the ratio of family income to federal poverty threshold in terms of FPL.

Reported ED/UCC visits in the past 12 months among persons with asthma differed by demographic characteristics (Figure 8) (Supplementary Table 13, https://stacks.cdc.gov/view/cdc/109086). The prevalence was higher among children (17.9%) than among adults (10.1%) (Supplementary Table 13, https://stacks.cdc.gov/view/cdc/109086 and was higher among women aged ≥18 years (11.5%) than among men aged ≥18 years (7.4%) (Supplementary Table 13, https://stacks.cdc.gov/view/cdc/109086). Among children, the prevalence did not differ by sex. Prevalence was highest among children aged 0–4 years (34.9%), followed by those aged 5–11 years (19.3%) and 12–17 years (11.4%). Among adults, no significant differences in ED/UCC visits were observed among age groups (Supplementary Table 13, https://stacks.cdc.gov/view/cdc/109086). Estimates of ED/UCC visits among AI/AN persons were not reliable because of the small sample size. Percentages of ED/UCC visits among all other racial and ethnic groups (i.e., Black, Asian, multiple race, and Hispanic, including Puerto Rican, Mexican, and other Hispanic) were higher than the percentage among White persons (Figure 8) (Supplementary Table 13, https://stacks.cdc.gov/view/cdc/109086).

The percentage of persons reporting ED/UCC visits within the past 12 months decreased as the ratio of income to poverty increased. The percentage with an ED/UCC visit was 18.1% among persons with family incomes <100% of the FPL, 14.4% among persons with family incomes 100% to <250% of the FPL, 9.6% among persons with family incomes 250% to <450% of the FPL, and 7.1% among persons with family incomes ≥450% of the FPL (Figure 8) (Supplementary Table 13, https://stacks.cdc.gov/view/cdc/109086).

#### Prevalence of Emergency Department and Urgent Care Center Visits by Geographic Location 

The percentage of persons reporting ED/UCC visits among all ages and among adults were significantly higher in the South (all ages: 14.2%; adults: 12.6%) than in the Northeast (all ages: 10.8%; adults: 8.8%) and the Midwest (all ages: 9.7%; adults: 7.9%) (Table 7). The percentage of reported ED/UCC visits among all ages and children with asthma were significantly higher in large MSAs (all ages: 13.1%; children: 20.1%) than in non-MSAs (all ages: 8.7%; children: 12.2%) (Table 7).

**FIGURE 9:**
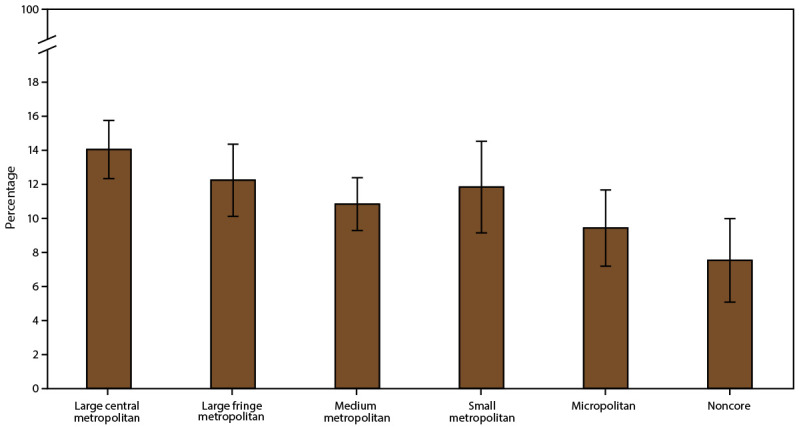
Prevalence of emergency department visits and urgent care center visits* because of asthma among persons with current asthma,^†^ by urban-rural classification^§^ — United States, 2016–2018 **Source:** CDC, National Center for Health Statistics, National Health Interview Survey. https://www.cdc.gov/nchs/nhis/index.htm * Prevalence is the proportion of the population with current asthma who reported having had one or more emergency department or urgent care center visits because of asthma in the past 12 months, with 95% confidence intervals indicated by error bars. ^†^ Includes persons who answered “yes” to the questions, “Have you ever been told by a doctor or other health professional that you had asthma?” and “Do you still have asthma?” ^§^ Large central metropolitan areas are counties in MSAs of ≥1 million population containing the principal city; large fringe metropolitan areas are counties in MSAs of ≥1 million population not containing the principal city; medium metropolitan areas are counties in MSAs of 250,000–999,999 population; small metropolitan areas are counties in MSAs of <250,000 population; micropolitan areas have an urban cluster population of 10,000–49,999; and noncore areas include nonmetropolitan areas that did not qualify as micropolitan, including those without an urban cluster population of at least 10,000.

Among urban-rural categories, the percentage of reported ED/UCC visits among persons of all ages with asthma was significantly higher in large central metropolitan areas (14.0%) than in micropolitan (9.4%) and noncore areas (7.5%) (Figure 9). Although percentages of reported ED/UCC visits among adults with asthma did not differ by urban-rural classification, the percentage among children with asthma was significantly higher in large central metropolitan areas (23.0%) than in noncore areas (10.2%) (Table 7).

Differences in ED/UCC visits by U.S. Census region and MSA category were significant for adults and all ages but not for children (Table 8). The percentage of reported ED/UCC visits in small MSAs was significantly higher in the South (all ages: 14.3%; adults: 12.3%) than in the Northeast (all ages: 7.9%; adults: 6.0%).

**TABLE 8:** Prevalence of emergency department and urgent care center visits* because of asthma in the past 12 months among all ages, children aged 0–17 years, and adults aged ≥18 years with current asthma,^†^ by U.S. Census region and metropolitan statistical area category**^§^** — United States, 2016–2018

U.S. Census region	Emergency department and urgent care center visits								
	Large MSA			Small MSA			Non-MSA		
	Weighted no.^¶^	Prevalence % (SE)	(95% CI)	Weighted no.^¶^	Prevalence % (SE)	(95% CI)	Weighted no.^¶^	Prevalence % (SE)	(95% CI)
**All ages**	**p = 0.47**			p = 0.01**			**p = 0.92**		
Northeast	392,354	12.5 (1.26)	(10.2–15.2)	118,629	7.9 (1.37)	(5.6–11.1)	35,263	8.6 (1.64)	(5.9–12.4)
Midwest	301,506	10.7 (1.27)	(8.4–13.5)	151,962	9.3 (1.16)	(7.2–11.8)	94,229	7.8 (1.90)	(4.8–12.4)
South	729,548	15.5 (1.20)	(13.3–18.0)	409,095	14.3 (1.46)	(11.7–17.5)	125,340	9.4 (1.40)	(7.0–12.5)
West	407,947	12.4 (1.74)	(9.3–16.2)	220,831	10.5 (1.03)	(8.7–12.7)	42,605	9.2 (2.08)	(5.9–14.2)
**Children**	p = 0.99			p = 0.33			p = 0.67		
Northeast	142,983	19.8 (3.47)	(13.8–27.5)	49,583	14.1 (3.28)	(8.8–21.8)	11,684	17.3 (6.40)^††^	(8.0–33.5)
Midwest	119,438	19.6 (3.44)	(13.7–27.2)	40,356	11.7 (3.04)	(6.9–19.1)	36,109	12.5 (3.83)^††^	(6.7–22.1)
South	250,223	20.7 (2.38)	(16.5–25.8)	156,857	19.5 (3.13)	(14.1–26.4)	30,203	9.8 (3.21)^††^	(5.0–18.1)
West	138,498	19.9 (3.23)	(14.3–27.0)	73,681	16.5 (3.57)	(10.6–24.7)	13,974	15.2 (5.31)^††^	(7.4–28.7)
**Adults**	p = 0.04**			p = 0.04**			p = 0.58		
Northeast	249,371	10.3 (1.33)	(8.0–13.2)	69,046	6.0 (1.17)	(4.1–8.8)	23,579	6.9 (1.56)	(4.4–10.7)
Midwest	182,067	8.2 (1.27)	(6.1–11.1)	111,606	8.6 (1.19)	(6.6–11.3)	58,120	6.3 (1.74)	(3.6–10.7)
South	479,325	13.7 (1.41)	(11.2–16.7)	252,238	12.3 (1.72)	(9.3–16.1)	95,137	9.2 (1.48)	(6.7–12.6)
West	269,449	10.3 (1.75)	(7.4–14.3)	147,150	8.9 (1.19)	(6.9–11.6)	28,631	7.7 (2.35)^††^	(4.2–13.8)

**Source:** CDC, National Center for Health Statistics, National Health Interview Survey. https://www.cdc.gov/nchs/nhis/index.htm

**Abbreviations:** CI = confidence interval; MSA = metropolitan statistical area; SE = standard error.

* Having had one or more emergency room or urgent care center visits because of asthma in the past 12 months among persons with current asthma.

^†^ Includes persons who answered “yes” to the questions, “Have you ever been told by a doctor or other health professional that you had asthma?” and “Do you still have asthma?”

**^§^** Large MSAs have a population of ≥1 million; small MSAs have a population of <1 million. Non-MSAs consist of persons not living in an MSA.

^¶^ National Health Interview Survey sample weights were used to adjust for nonresponse, poststratification, and probability of selection to provide estimates for the intended U.S. populations.

** Statistically significant by chi-square test, p<0.05.

^††^ Relative SE = 30%–50%; estimate is unreliable.

### Asthma Mortality Rate 

During 2016–2018, the asthma mortality rate was 10.8 per million among all ages. The rate was almost four times higher among adults (13.2 per 1 million) than among children (2.7 per 1 million) (Table 9).

**TABLE 9:** Asthma mortality rate* among all ages, children aged 0–17 years, and adults aged ≥18 years, by geographic area — United States, 2016–2018

Characteristic	Asthma mortality rate								
	All ages			Children			Adults		
	No.	Rate per million (SE)	(95% CI)	No.	Rate per million (SE)	(95% CI)	No.	Rate per million (SE)	(95% CI)
**Total**	**10,523**	**10.8 (0.11)**	**(10.6–11.0)**	**586**	**2.7 (0.11)**	**(2.4–2.9)**	**9,936**	**13.2 (0.13)**	**(12.9–13.4)**
**U.S. Census region**									
Northeast	1,978	11.7 (0.26)	(11.2–12.2)	100	2.8 (0.28)	(2.3–3.4)	1,877	14.0 (0.32)	(13.4–14.7)
Midwest	2,315	11.3 (0.24)	(10.9–11.8)	157	3.4 (0.27)	(2.8–3.9)	2,158	13.7 (0.29)	(13.1–14.2)
South	3,649	9.8 (0.16)	(9.5–10.2)	250	2.9 (0.19)	(2.6–3.3)	3,399	11.9 (0.20)	(11.5–12.3)
West	2,581	11.1 (0.22)	(10.7–11.6)	79	1.5 (0.17)	(1.2–1.8)	2,502	14.0 (0.28)	(13.5–14.6)
**MSA category^†^**									
Large MSA	5,597	10.3 (0.14)	(10.0–10.5)	348	2.8 (0.15)	(2.5–3.1)	5,248	12.5 (0.17)	(12.1–12.8)
Small MSA	3,212	11.0 (0.19)	(10.6–11.3)	164	2.5 (0.19)	(2.1–2.9)	3,048	13.4 (0.24)	(13.0–13.9)
Non-MSA	1,714	12.4 (0.30)	(11.8–13.0)	74	2.4 (0.28)	(1.9–3.1)	1,640	15.2 (0.38)	(14.5–15.9)
**Urban-rural classification^§^**									
Large central metropolitan	3,454	11.5 (0.20)	(11.1–11.9)	229	3.4 (0.22)	(2.9–3.8)	3,225	13.8 (0.24)	(13.4–14.3)
Large fringe metropolitan	2,143	8.8 (0.19)	(8.4–9.2)	119	2.1 (0.19)	(1.7–2.5)	2,023	10.8 (0.24)	(10.3–11.2)
Medium metropolitan	2,223	10.9 (0.23)	(10.4–11.3)	116	2.5 (0.23)	(2.0–2.9)	2,107	13.4 (0.29)	(12.8–14.0)
Small metropolitan	989	11.1 (0.35)	(10.4–11.8)	48	2.5 (0.35)	(1.8–3.2)	941	13.6 (0.44)	(12.7–14.4)
Micropolitan	957	11.7 (0.38)	(11.0–12.4)	46	2.5 (0.37)	(1.9–3.4)	911	14.3 (0.47)	(13.4–15.2)
Noncore	757	13.4 (0.49)	(12.4–14.4)	28	2.3 (0.43)	(1.5–3.3)	729	16.5 (0.61)	(15.3–17.7)

**Source:** CDC, National Center for Health Statistics, National Vital Statistics System, CDC WONDER. https://wonder.cdc.gov/

**Abbreviations:** CI = confidence interval; MSA = metropolitan statistical area; SE = standard error.

* Underlying cause of death is asthma; includes *International Classification of Diseases, Tenth Revision* codes J45–J46.

^†^ Large MSAs have a population of ≥1 million; small MSAs have a population of <1 million. Non-MSAs consist of persons not living in an MSA.

**^§^** Large central metropolitan areas are counties in MSAs of ≥1 million population containing the principal city; large fringe metropolitan areas are counties in MSAs of ≥1 million population not containing the principal city; medium metropolitan areas are counties in MSAs of 250,000–999,999 population; small metropolitan areas are counties in MSAs of <250,000 population; micropolitan areas have an urban cluster population of 10,000–49,999; and noncore areas include nonmetropolitan areas that did not qualify as micropolitan, including those without an urban cluster population of at least 10,000.

#### Trends in Asthma Mortality Rates

**FIGURE 10:**
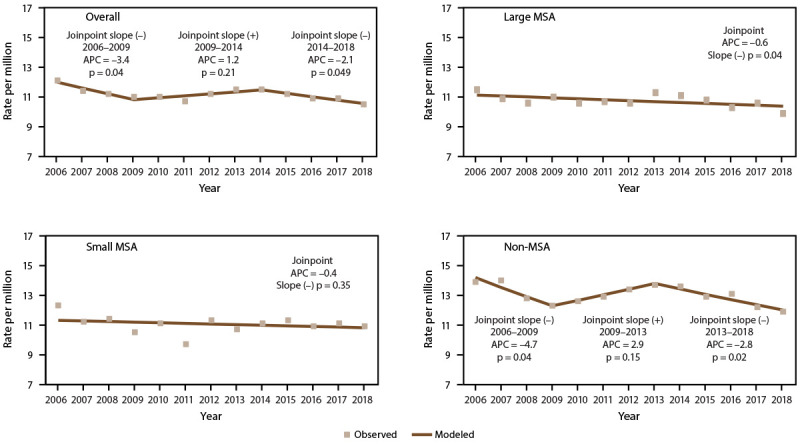
Asthma mortality rate,* by year and metropolitan statistical area category^†^ — United States, 2006–2018 **Source:** CDC, National Center for Health Statistics, National Health Interview Survey. https://www.cdc.gov/nchs/nhis/index.htm **Abbreviations:** APC = annual percent change; MSA = metropolitan statistical area. * Underlying cause of death is asthma; includes *International Classification of Diseases, Tenth Revision* codes J45–J46. Statistical significance set at p<0.05. ^†^ Large MSAs have a population of ≥1 million; small MSAs have a population of <1 million. Non-MSAs consist of persons not living in an MSA.

The asthma mortality rates overall (Figure 10) and among adults decreased significantly (2006–2009 overall: APC = −3.4; 2006–2009 adults: APC = −3.8; 2014–2018 overall: APC = −2.1), particularly in large MSAs and non-MSAs (Supplementary Tables 20 and 23, https://stacks.cdc.gov/view/cdc/109086). Asthma mortality rates among children remained stable (Supplementary Table 23, https://stacks.cdc.gov/view/cdc/109086).

#### Differences in Asthma Mortality Rates by Demographic Characteristics

**FIGURE 11:**
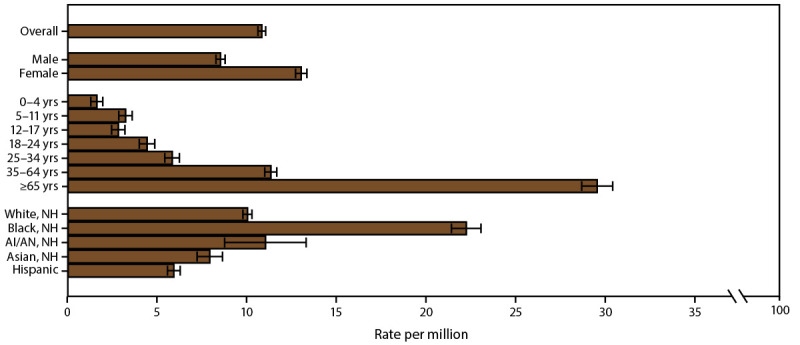
Asthma mortality rate,* by sex, age group, and race/ethnicity — United States, 2016–2018 **Source:** CDC, National Center for Health Statistics, National Health Interview Survey. https://www.cdc.gov/nchs/nhis/index.htm **Abbreviations:** AI/AN = American Indian or Alaska Native; NH = non-Hispanic. * Underlying cause of death is asthma; includes *International Classification of Diseases, Tenth Revision* codes J45–J46; 95% confidence intervals are indicated by error bars.

Asthma mortality rates differed significantly by demographic characteristics (Figure 11) (Supplementary Table 18, https://stacks.cdc.gov/view/cdc/109086). The rate was higher among adults (13.2 per million) than among children (2.7 per million) and higher among females (13.0 per million) than among males (8.5 per million) (Figure 11) (Supplementary Table 18, https://stacks.cdc.gov/view/cdc/109086). The difference in the mortality rate between males and females varied by age group. Among children, boys had a higher rate than girls (3.1 compared with 2.2). Among adults, women had a higher rate than men (16.0 compared with 10.2) (Figure 11) (Supplementary Table 18, https://stacks.cdc.gov/view/cdc/109086). Asthma mortality rates increased with age, from 1.6 per 1 million among children aged 0–4 years to 29.5 per million among adults aged ≥65 years (Figure 11). Asthma mortality rates were significantly higher among Black persons (22.2 per million) and significantly lower among Asian persons (7.9 per million) and Hispanic persons (5.9 per million) than among White persons (10.0 per million). The rate for AI/AN persons (11.0) was similar to the rate for White persons (Figure 11) (Supplementary Table 18, https://stacks.cdc.gov/view/cdc/109086).

#### Asthma Mortality by Geographic Location 

The asthma mortality rate (per 1 million) was significantly higher among persons in the Northeast (all ages: 11.7; adults: 14.0), the Midwest (all ages: 11.3; adults: 13.7), and the West (all ages: 11.1; adults: 14.0) than among those in the South (all ages: 9.8; adults: 11.9) (Table 9). Among children, the rate (per 1 million) was significantly higher among those in the Northeast (2.8), the Midwest (3.4), and the South (2.9) than among those in the West (1.5).

Although the rate by MSA category did not differ among children, the rate differed significantly among persons of all ages and adults. The rate was higher in non-MSAs (all ages: 12.4; adults: 15.2), followed by small MSAs (all ages: 11.0; adults: 13.4) and large MSAs (all ages: 10.3; adults: 12.5) (Table 9).

**FIGURE 12:**
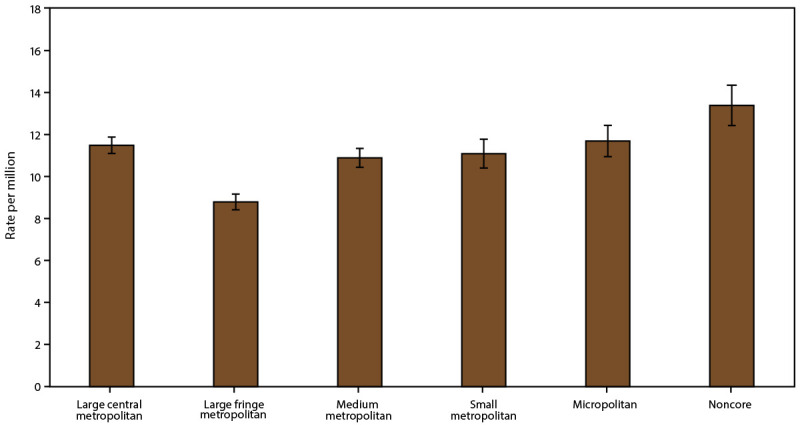
Asthma mortality rate,* by urban-rural classification^†^ — United States, 2016–2018 **Source:** CDC, National Center for Health Statistics, National Health Interview Survey. https://www.cdc.gov/nchs/nhis/index.htm **Abbreviation:** MSA = metropolitan statistical area. * Underlying cause of death is asthma; includes *International Classification of Diseases, Tenth Revision* codes J45–J46; 95% confidence intervals are indicated by error bars. ^†^ Large central metropolitan areas are counties in MSAs of ≥1 million population containing the principal city; large fringe metropolitan areas are counties in MSAs of ≥1 million population not containing the principal city; medium metropolitan areas are counties in MSAs of 250,000–999,999 population; small metropolitan areas are counties in MSAs of <250,000 population; micropolitan areas have an urban cluster population of 10,000–49,999; and noncore areas include nonmetropolitan areas that did not qualify as micropolitan, including those without an urban cluster population of at least 10,000.

Asthma mortality rates among persons of all ages and adults were significantly higher in noncore areas (13.4 and 16.5, respectively) and lower in large fringe metropolitan areas (8.8 and 10.8, respectively) than in other urban-rural categories. The rate among children did not differ by urban-rural classification (Table 9) (Figure 12).

Asthma mortality rates by MSA category differed significantly among U.S. Census regions (Table 10). The rate in large MSAs was higher in the Northeast (all ages:12.0; adults: 14.3) and the Midwest (all ages: 11.6; adults: 13.8) than in the South (all ages: 8.9; adults: 10.9) and the West (all ages: 9.8; adults: 12.2). For children, the rate in large MSAs was higher in the Northeast (3.3), the Midwest (4.2), and the South (2.7) than in the West (1.6) (Table 10).

**TABLE 10:** Asthma mortality rate* among all ages, children aged 0–17 years, and adults aged ≥18 years, by U.S. Census region and metropolitan statistical area category — United States, 2016–2018

U.S. Census region	Asthma mortality rate								
	Large MSA^†^			Small MSA^†^			Non-MSA^†^		
	No.	Rate per million (SE)	(95% CI)	No.	Rate per million (SE)	(95% CI)	No.	Rate per million (SE)	(95% CI)
**All ages**									
Northeast	1,362	12.0 (0.33)	(11.4–12.7)	468	11.2 (0.52)	(10.2–12.2)	148	10.8 (0.89)	(9.1–12.5)
Midwest	1,153	11.6 (0.34)	(10.9–12.3)	607	10.2 (0.41)	(9.3–11.0)	555	12.3 (0.52)	(11.3–13.3)
South	1,688	8.9 (0.22)	(8.5–9.4)	1,248	10.2 (0.29)	(9.6–10.8)	713	12.1 (0.45)	(11.2–12.9)
West	1,394	9.8 (0.26)	(9.2–10.3)	889	12.9 (0.43)	(12.0–13.7)	298	14.7 (0.85)	(13.0–16.4)
**Children**									
Northeast	80	3.3 (0.37)	(2.7–4.2)	17	—^§^	—^§^	—^§^	—^§^	—^§^
Midwest	97	4.2 (0.43)	(3.4–5.1)	38	2.8 (0.46)	(2.0–3.9)	22	2.2 (0.47)	(1.4–3.3)
South	120	2.7 (0.25)	(2.2–3.2)	87	3.2 (0.34)	(2.5–3.9)	43	3.3 (0.50)	(2.4–4.4)
West	51	1.6 (0.22)	(1.2–2.1)	22	1.3 (0.28)	(0.8–2.0)	—^§^	—^§^	—^§^
**Adults**									
Northeast	1,281	14.3 (0.40)	(13.5–15.1)	451	13.6 (0.64)	(12.4–14.9)	145	13.1 (1.09)	(11.0–15.2)
Midwest	1,056	13.8 (0.42)	(13.0–14.6)	569	12.3 (0.52)	(11.3–13.3)	533	15.2 (0.66)	(13.9–16.5)
South	1,568	10.9 (0.27)	(10.3–11.4)	1,161	12.2 (0.36)	(11.5–12.9)	670	14.6 (0.56)	(13.5–15.7)
West	1,343	12.2 (0.33)	(11.5–12.8)	867	16.6 (0.56)	(15.5–17.7)	292	18.6 (1.09)	(16.5–20.7)

**Source:** CDC, National Center for Health Statistics, National Vital Statistics System, CDC WONDER. https://wonder.cdc.gov/

**Abbreviations:** CI = confidence interval; MSA = metropolitan statistical area; SE = standard error.

* Underlying cause of death is asthma; includes *International Classification of Diseases, Tenth Revision* codes J45–J46.

^†^ Large MSAs have a population of ≥1 million; small MSAs have a population of <1 million. Non-MSAs consist of persons not living in an MSA.

^§^ Suppressed because N ≤19 persons.

The asthma mortality rate in small MSAs was higher in the West (all ages: 12.9; adults: 16.6) than in the Midwest (all ages: 10.2; adults: 12.3), the South (all ages: 10.2; adults: 12.2), and the Northeast (adults: 13.6). Comparison of rates among children was not possible because of suppressed values (Table 10).

Among persons of all ages and adults, the rates in non-MSAs were higher in the West (14.7 and 18.6, respectively) than in the Northeast (10.8 and 13.1, respectively) and in the South (12.1 and 14.6, respectively). Comparison of rates among children was not possible because of suppressed values (Table 10).

## Discussion

This report assessed prevalence of current asthma, asthma attacks, ED/UCC visits, and asthma-related mortality rates by year, demographics, poverty level (except for mortality), and geographic area, including urban-rural classifications, in the United States. Rural health has become increasingly important in monitoring health in the United States and in health equity (*16*,*17*). Measuring asthma indicators in geographic areas provides information on progress toward HHS *Healthy People 2030* strategic goals of reducing health disparities (*19*) and can aid public health programs in directing resources and interventions to improve the health of persons with asthma.

As previously described (*5*,*37*), although asthma prevalence decreased among children during 2006–2018 (Supplementary Table 23, https://stacks.cdc.gov/view/cdc/109086), the trend remained stable among adults. In all MSA categories, the prevalence of asthma attacks and ED/UCC visits decreased among all ages, and asthma mortality decreased among adults, indicating improvement in health outcomes and health care use over time. The availability of new evidence-based strategies in the medical management of asthma might have helped clinicians to optimize treatment strategies and apply current treatment guidelines (*38*,*39*), which might have contributed to these decreases. Despite these decreases, disparities persist.

Previous studies have shown that asthma prevalence differs by demographic factors and income (*1*,*30*). Differences in prevalence of asthma, asthma attacks, ED/UCC visits, and mortality rates were observed across MSA categories (Supplementary Tables 2, 8, 14, and 19, https://stacks.cdc.gov/view/cdc/109086). Asthma was more prevalent among certain subpopulations (e.g., boys aged <18 years, women aged ≥18 years, children aged 12–17 years, Black persons, persons of multiple races, Puerto Rican persons, and persons with a low ratio of income to poverty). Demographic disparities in asthma prevalence have persisted over time (*5*,*30*,*37*). Increased awareness among clinicians could improve medical management of asthma for persons in disproportionately affected subpopulations. Asthma attacks were more prevalent among children, women, persons of multiple races, and those with a low ratio of income to poverty. ED/UCC visits were more prevalent among children, Black persons, Asian persons, persons of multiple races, Hispanic persons, and persons having a low ratio of income to poverty. The higher prevalence of asthma attacks and ED/UCC visits among children, especially those aged 0–4 years, compared with adults might be explained by their susceptibility to respiratory infections and other environmental hazards (*40*). Persons with low incomes and in non-White racial groups are shown to have worse health outcomes (*5*,*10*,*12*,*24*) because of their risk factors, environmental exposures, and reduced access to quality health care (*12*).

Mortality rates were higher among adults than among children, among boys aged <18 years than among girls aged <18 years, among women aged ≥18 years than among men aged ≥18 years, and among Black persons than among White persons. Asthma-related mortality rates also increased with age. One study found significant racial and sex differences in asthma mortality rates (*41*).

Socioeconomic status, particularly income, is an important determinant of health among persons with asthma (*14*,*25*,*30*). Persons with inadequate resources might face barriers to accessing quality health care, resulting in unmet health care needs, worsening health, and increased ED visits and hospitalizations (*15*,*19*).

The prevalence of current asthma, asthma attacks, and ED/UCC visits and asthma mortality rates differed by geographic location (i.e., U.S. Census region, MSA category, urban-rural classification, and state). Variations in asthma outcomes by geographic areas might be explained by differences in sociodemographic characteristics and health care policy that might determine health care access and specialty care (*8*,*10*,*12*,*15*). Asthma prevalence varied by state, with a higher prevalence in the Northeast than the South and the West and a higher prevalence in small MSAs than in large MSAs. Among small MSAs, prevalence was higher in the Northeast than in the South. In large MSAs, the mortality rate was higher in the Northeast and the Midwest than in the South and the West. One study found that residents of large central metropolitan areas lacked health insurance, and residents of those areas in the Northeast had worse health outcomes (*8*). An area referred to as the Northeast Mid-Atlantic Asthma Belt (from Greensboro, North Carolina, to Boston, Massachusetts) has been shown to have a higher prevalence of asthma, possibly due to poverty, poor air quality, and less access to specialists (*42*). Four states had differences in prevalence of asthma attacks by MSA category, including New York, North Dakota, Ohio, and Tennessee.

The prevalence of ED/UCC visits was higher in the South and among large MSAs nationwide. Specifically, asthma-related ED/UCC visits were higher in large central metropolitan areas than in nonmetropolitan areas, including micropolitan and noncore areas. Urban residents have greater disparities in ongoing sources of care (*43*) and might experience adverse environmental exposures and stressors (*20*,*21*), which could explain higher ED/UCC use. Numbers of ED/UCC visits might be lower in noncore areas because the distance to receive care might be greater or because of a lack of nearby hospitals due to rural hospital closures (*18*) and lack of transportation (*9*). The South has the largest poverty gap between rural and urban residents (*44*) and the highest rate of potentially preventable adult hospitalizations for chronic disease (*45*). Rural disparities could be a result of economic hardship and lack of access to health care (*44*). Asthma mortality rates were higher in non-MSAs, specifically in noncore areas, consistent with findings from another study (*15*), which also identified disparities in excess deaths in rural areas, in which the residents are older, poorer, and sicker than residents of urban areas. Unhealthy behaviors and certain social circumstances have also been identified as contributors to early mortality among rural residents (*12*).

This report is fifth in a series of asthma surveillance summaries (*27*–*30*), with an additional focus on geographic areas, including MSA categories, urban-rural classification, and location by state. The four asthma indicators (i.e., prevalence of current asthma, asthma attacks, ED/UCC visits, and asthma-associated deaths) were assessed across various geographic areas (including four U.S. Census regions, MSA categories, a six-level urban-rural classification, and the 50 states and Washington, DC) by demographic characteristics and poverty level (except for mortality). The findings in this report provide insights that might help to direct public health resources, policy, and interventions to improve health of persons with asthma.

## Limitations

This findings in this report are subject to at least five limitations. First, NHIS data are self-reported, and self-reports are subject to biases (e.g., social desirability and selective recall), possibly resulting in misclassification. However, NHIS has been collecting data on a broad range of health topics since 1957 (*33*). Second, as in the previous asthma surveillance summaries, this report used descriptive statistics to define the prevalence of asthma in the population. The associations and differences are not necessarily causal, and variables other than those considered might be responsible for certain observed differences. Third, urban-rural and MSA categories are based on county-level population characteristics, not county size or zip code, and might have resulted in misclassifications. Fourth, Joinpoint trend analysis using aggregated survey data might have resulted in small variance estimates for the slopes, which could have affected statistical significance. Finally, analyses of group differences did not adjust for multiple comparisons.

## Future Directions

Periodic assessments and reports on asthma-related health outcomes and health care use in the population by various geographic locations are essential. The findings can be used to better implement strategies to improve health and quality of life among persons with asthma.

## Conclusion

The overall prevalence of current asthma remains stable, although disparities persist. The prevalence of asthma attacks and ED/UCC visits, as well as asthma mortality rates, decreased over time. Asthma indicators differed by age, sex, race/ethnicity, poverty level, and geographic location. The prevalence of asthma was higher in small MSAs than in large MSAs and in the Northeast than in the South and the West. The prevalence of ED/UCC visits was higher in the South than in the Northeast and the Midwest and in large MSAs than in non-MSAs. Asthma mortality rates were higher in the Northeast, the Midwest, and the West than in the South and in non-MSAs, especially in noncore areas. Geographic variations in demographic characteristics, environmental factors, economy, and health care policies might explain variations in asthma outcomes and health care use by geographic location. Findings from this report can aid public health programs in directing resources and interventions to improve asthma-related health outcomes and health care use, developing strategic goals, and achieving the CCARE initiative to reduce childhood asthma hospitalizations and ED visits.
